# Stromatolitic Mounds in Tidal‐Facies Sandstones of the Paleoarchean Moodies Group (Barberton Greenstone Belt, Eswatini)

**DOI:** 10.1111/gbi.70020

**Published:** 2025-05-02

**Authors:** Sebastian Reimann, Martin Homann, Deon J. Janse van Rensburg, Michael Wiedenbeck, Christian Hallmann, Runa Antony, Christoph Heubeck

**Affiliations:** ^1^ Department of Geosciences Friedrich‐Schiller‐Universität Jena Jena Germany; ^2^ University College London London UK; ^3^ GFZ—German Research Center for Geosciences Telegrafenberg Potsdam Germany; ^4^ University of Potsdam Potsdam Germany

## Abstract

Shallow‐marine environments are thought to have been pivotal to the spreading, perhaps even the origin, of early life on Earth. The shallow‐marine Archean sedimentary record of early life, however, is biased towards carbonates; nearshore siliciclastic environments have not received proportional attention. Here we describe densely laminated, silicified and dolomitized fossil calcareous mounds in tidal‐facies sandstones of the Archean Moodies Group (ca. 3.22 Ga) in the Barberton Greenstone Belt, Eswatini. They vary between (1) cm‐ to dm‐scale, isolated, club‐ to pedestal‐shaped, nodular mounds on top of and within the conduits of fluid‐escape structures, and (2) mm‐ to cm‐scale, undulatory and wavily laminated structures, interbedded with well‐bedded silt‐ and sandstones. Geochemical indicators of a possible biogenic origin were largely obliterated by local hydrothermal alteration and regional lower‐greenschist‐facies metamorphism: In situ SIMS δ^13^C_carb_ isotope analyses from several traverses across the best‐preserved laminae of a mound and δ^34^S_VCDT_ values from diagenetic rims of nearby detrital pyrite grains yield ambiguous isotopic evidence about biologic processing; TOC of putative laminae is too low to measure δ^13^C_org_, and Raman spectroscopy of finely dispersed carbonaceous particles and of kerogenous laminae indicate mean maximum metamorphic temperature of ca. 500°C. Textural and regional evidence, however, suggests that the carbonate laminae represent metabolic products of microbial communities that took advantage of sand volcanoes from which nutrient‐rich fluids erupted episodically. We base this inference on the habitable depositional setting on a wave‐ or current‐swept photic‐zone tidal platform, the stromatolitic morphologies in two and three dimensions, the occurrence of in‐situ kerogen, the carbonate mineralogy, and the presence of comparable mound structures elsewhere in the Moodies Group. Although the metabolic strategies utilized by the microorganisms remain unknown, this occurrence places a novel ecologic niche in the Paleoarchean microbial colonization of coastal regions.

## 1 Introduction and Geologic Setting

Over the course of early Earth history, microbes adapted to the diverse surface conditions by developing manifold metabolic strategies (e.g., Guy et al. 2012; Nabhan et al. 2016; Olson and Straub 2016; Ménez 2020) which allowed them to colonize various environments. These, in turn, affected the morphology of sedimentary structures formed by early life as a function of, for example, the presence and nature of fluids and nutrients, the intensity and variability of solar radiation, temperature, current and wave energy, the degree of physical abrasion, and salinity. Following the rise of atmospheric oxygen, some of these metabolic strategies retreated from the surface to niche settings (e.g., Stüeken and Buick 2018).

Over time, solitary microorganisms colonizing benthic aqueous environments evolved into complex microbial communities that formed stromatolites (e.g., Russell and Hall 2006; Hickman‐Lewis and Westall 2021). Following Viehmann et al. (2020), we define stromatolites as “organo‐sedimentary structures formed by the incidental induction of mineral precipitation within or on microbial biofilms with, or without, trapping and binding of ambient sediments”. The oldest well‐described stromatolites occur in the Dresser Formation of the North Pole Dome (ca. 3.48 Ga; Djokic et al. 2021 and references therein) and in the Strelley Pool Chert (ca. 3.34 Ga; Brasier et al. 2006; Wacey et al. 2006, 2008; Duda et al. 2016; Viehmann et al. 2020 and references therein) of the Pilbara craton, Australia. Lepot (2020) examined both locations in the context of potential abiotic sources for these siliceous structures.

Our understanding of the mechanisms that establish and support microbial growth and shape their morphology is hampered by diverse processes that influence the production of organic matter (Bosak et al. 2012), by motility, which redistributes biomass (e.g., Walter et al. 1976), and by respiration and fermentation, which remove biomass (Stal and Moezelaar 1997; Moorhead et al. 2013). The spatial distribution and temporal variation of these processes can produce morphologically diverse fossil structures. Since virtually all of the limited Paleoarchean sedimentary strata capable of preserving fossilized life forms are strongly altered by metamorphism and deformation, exceptional conditions are necessary to preserve convincing products of former metabolic activities (e.g., Oró et al. 1974; Sugitani et al. 2007; Wacey 2009; Homann 2019).

**FIGURE 1:**
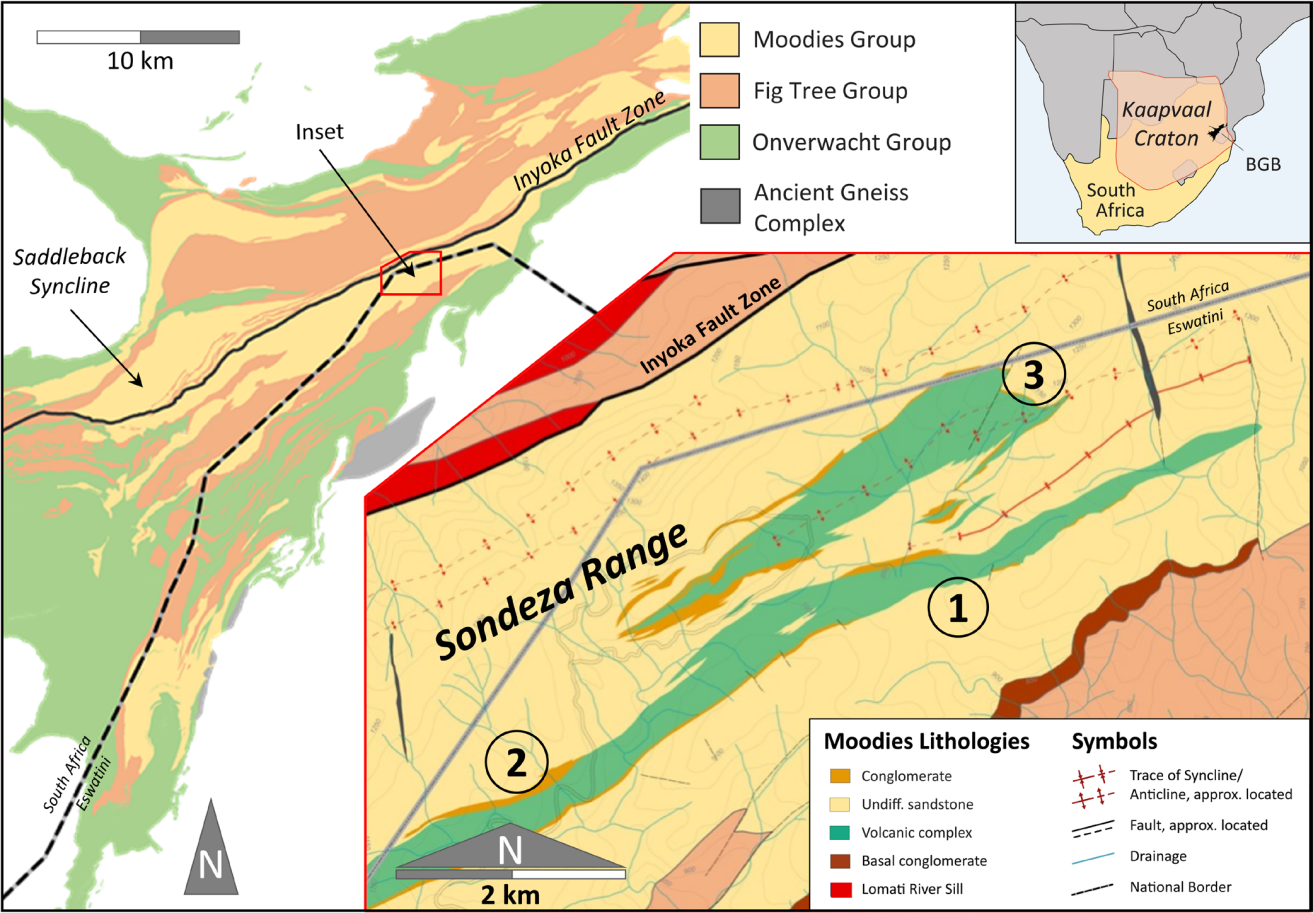
Simplified geological map of the Barberton Greenstone Belt (left). Red polygon shows the location of the study area. Inset shows geological map of parts of the Sondeza Range with the locations of numbered investigated sites near the Moodies volcanic complex (Figure 2). Putative microbial mats containing traces of likely kerogenous organic matter are preserved at Site 1, calcareous mounds at Sites 2 and 3.

The ca. 3.22 Ga Moodies Group (Heubeck et al. 2013; Heubeck 2019) of the Barberton Greenstone Belt (BGB; Byerly et al. 2019) in South Africa and Eswatini is such an exceptionally preserved remnant of Paleoarchean shallow‐water and terrestrial environments. Moodies Group strata reach a thickness of up to 3.7 km and generally show remarkably good preservation and excellent outcrop conditions, allowing high temporal resolution and detailed correlation of facies relationships along and across strike (e.g., Homann et al. 2015; Stutenbecker et al. 2019; Janse van Rensburg et al. 2021; Zametzer et al. 2023). Stratigraphic units in the Sondeza Range region of northernmost Eswatini are contiguous with the better investigated Moodies Group strata in South Africa south of the Inyoka Fault (Figure 1). Moodies Group strata throughout the BGB are dominated by fine‐ to coarse‐grained, quartz‐rich sandstones with subordinate conglomerates, siltstones, lavas, and tuffs, mostly deposited in terrestrial to shallow‐marine settings (Heubeck 2019). They experienced several phases of deformation at lower greenschist‐facies metamorphism (Toulkeridis et al. 1998; Lowe et al. 1999; Tice et al. 2004; Schmitz and Heubeck 2021). Among numerous sedimentary structures indicating shallow‐water, current‐ and wave‐dominated conditions, Moodies Group strata in the central BGB preserve abundant kerogenous laminae in tidal‐facies sandstones, which form cm‐scale bulbous buildups, tufts, and silicified cavity fills, likely representing fossilized microbial mats (Heubeck 2009; Gamper et al. 2012; Homann et al. 2015, 2016, 2018). Associated small carbonate mounds may be hybrid organic–anorganic precipitates (Heubeck et al. 2023); the setting of abundant fluid‐escape structures disrupting the kerogenous laminae is speculative (Stengel et al. 2024).

Here we describe and interpret partially silicified carbonate mounds of likely microbial origin of the Moodies Group that occur within and on top of syndepositional fluid‐escape structures. This association is novel for Archean strata and adds a new aspect of ecosystem complexity to shallow‐water habitats on early Earth.

## 2 Methods

**FIGURE 2:**
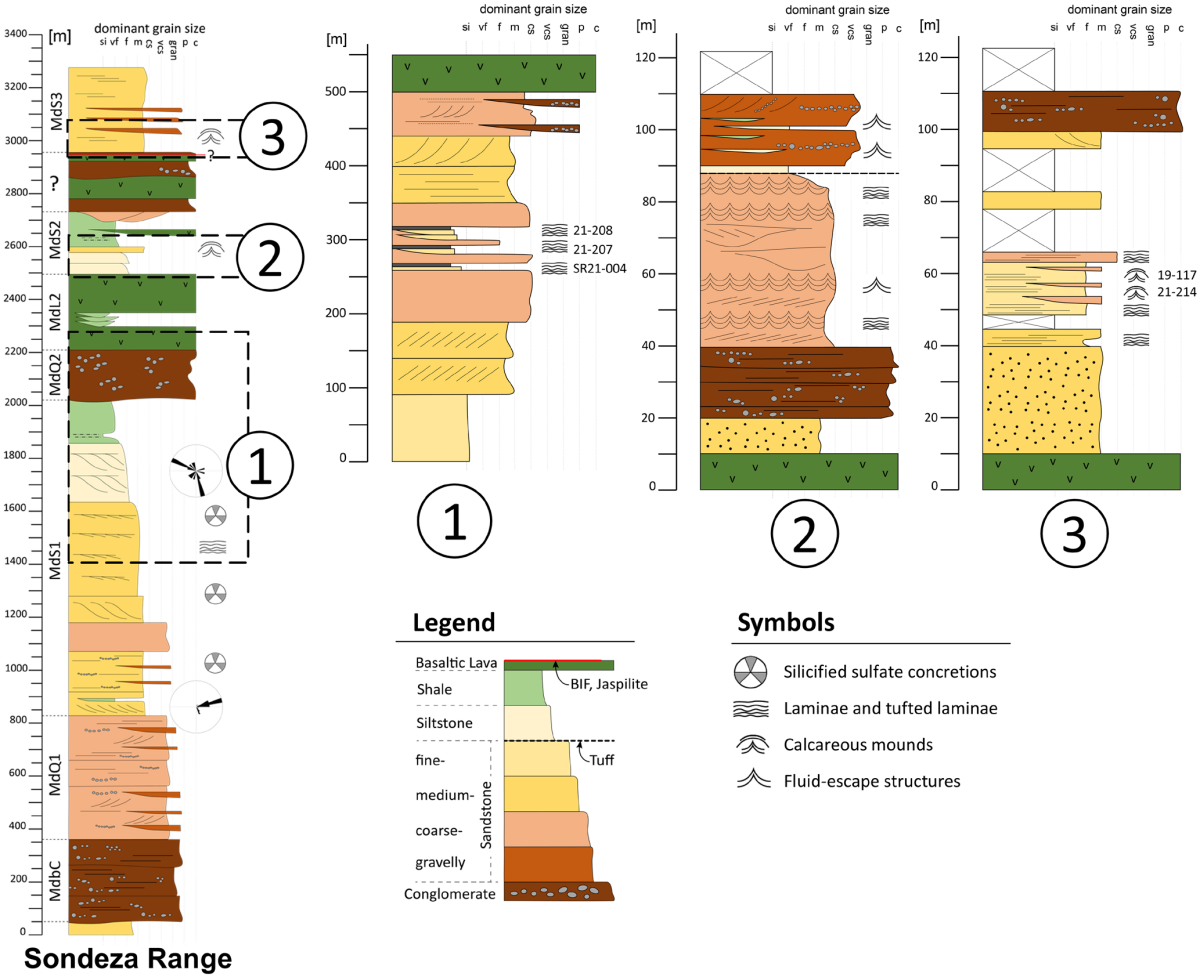
(Left) Generalized stratigraphic column of the Sondeza Range. The stratigraphic succession below the Moodies volcanic complex (correlatable to unit MdL2 of Anhaeusser (1978) in adjacent South Africa) can be readily interpreted using standard Moodies Group stratigraphic subdivisions. (right) Stratigraphic columns of Sites 1 to 3. At midsection, Site 1 shows undulatory, possibly carbonaceous laminations in several sandstone beds which are laterally traceable over several hundred m. At Site 2, calcareous laminations and mounds in thin siltstone beds are interbedded with coarse‐grained sandstone. At Site 3, calcareous laminations and mounds form dm‐scale structures in medium‐ to coarse‐grained sandstone.

We mapped parts of the Sondeza Range in Eswatini and sampled key locations (Figures 1 and 2; Table 1). Slabbing, photographic and microscopic documentation, line‐drawing, 3‐D modeling, micro‐X‐ray fluorescence (μ‐XRF), and Raman spectroscopy were performed at the Department of Geosciences, University of Jena.

**TABLE 1:** Coordinates of sample locations (WGS 1984, EPSG: 4326).

Sample no.	Coordinates	
	Long.	Lat.
19‐117	31.347348	−25.735763
21‐207	31.340247	−25.752567
21‐208	31.340307	−25.752816
21‐214	31.346737	−25.735643
SR21‐004	31.340337	−25.752815

Thirteen 30‐μm‐thick polished thin sections, oriented perpendicular to bedding, were studied to determine the petrography, mineralogy, and morphology of carbonate mounds, laminae, and host strata. High‐resolution photographs were made using a Keyence VHX‐6000 digital microscope. Four 1‐in.‐diameter, ca. 5 cm thick plugs were drilled from well‐preserved structures of sample 19‐117. These were cut into six or seven slices, each < 5 mm thick, embedded in epoxy, and polished on one side (Figure S3). High‐resolution element intensity maps of surfaces perpendicular to bedding were generated using a Bruker M4 Tornado μ‐XRF scanner with ca. 20 μm spatial resolution (Flude et al. 2017). We acquired Raman spectroscopy data using a Horiba LabRam Evolution system. Data were collected over the range of 400–1900 cm^−1^ using a 600 lines/mm spectrometer grating and a CCD detector. A Neodymium‐YAG laser at 532 nm was used as the light source; optical filters reduced the laser power to < 0.4 mW. Raman backscattering was recorded over an integration time of 50 s and 2 repetitions for each measured point to reduce fluorescence. Ten to fifty measurements were performed for each sample to ensure reproducibility. Organic targets were analyzed by a 2 μm‐diameter spot using a 50× and 100× objective. We used the Fityk curve‐fitting and analysis software (Wojdyr 2010) to do a Gaussian baseline correction and extract peak parameters in order to calculate peak paleotemperatures (Beyssac et al. 2002; Lünsdorf et al. 2014). We used a JEOL JXA‐8230 electron microprobe to obtain back‐scattered electron (BSE) images and wavelength‐dispersive (WDX) mineral analyses of pyrite grains (maps and total element measurements). Operating conditions were set to an accelerating voltage of 15 kV, a beam current of 15 nA, and a beam diameter of 1 μm. Wavelength‐dispersive X‐ray spectrometers were used to measure the concentrations of As (Kα X‐ray lines), Ni (Kα), Fe (Kα), S (Kα), Au (Kα), Cu (Kα) and Co (Kα) each with 20 s counting time.

In order to investigate biological processing, we determined 195 values of δ^34^S_VCDT_ (Vienna Canyon Diablo troilite) from 22 detrital pyrite grains in four mounts from the margins of two carbonate mounds and from one lamina. Measurements were made using the Cameca 1280‐HR secondary ion mass spectrometry (SIMS) instrument at the Deutsches GeoForschungsZentrum Potsdam. A single SIMS analysis involved 80 s of data acquisition in FC‐FC (Faraday cup) multicollection mode using a test portion mass of ~300 pg. Four 25.4 mm‐diameter mounts were prepared, with each mount including a millimeter‐size piece of the Balmat reference pyrite, embedded using epoxy in a small well at the center of each sample. This reference material was assigned a δ^34^S_VCDT_ value of 15.1‰ (Crowe and Vaughan 1996). An absolute value of ^34^S/^32^S = 0.044163 (Ding et al. 2001) was used as the zero point of the δ^34^S_VCDT_ scale. All mounts were cleaned with ethanol and gold‐coated before analyses. After correcting for small time‐dependent instrumental drift, the analytical session yielded a repeatability of ±0.11‰ (1 standard deviation, s.d.). A careful evaluation of the data set indicates that our individual analyses are reliable at a total analytical uncertainty level of better than ±0.48‰ (1 s.d.) in which the dominant source of uncertainty comes from the assigned uncertainty for the bulk characterization of the Balmat pyrite (Crowe and Vaughan 1996).

The same SIMS instrument was also used to measure 104 values of δ^13^C_PDB_ of calcite and dolomite mineral grains along four traverses on mount 19‐117‐P1‐I. A single analysis involved 80 s of data acquisition in FA‐FA multicollection mode. Calcite calibrations were based on the reference material UWC‐3 with an assigned a δ^13^C_PDB_ value of −0.91‰ (Kozdon et al. 2009). Dolomite calibrations were based on the reference material UW‐6620 with an assigned δ^13^C_PDB_ value of 0.84‰ (Śliwiński et al. 2015). The zero‐point of the PDB scale was set to an absolute value of ^13^C/^12^C = 0.011246639 (Craig 1957). After correcting for a small time‐dependent instrumental drift, the analyses yielded a repeatability of ±0.24‰ (1 s.d.). A careful evaluation of the data set indicates that our individual analyses are reliable at a total analytical uncertainty level of better than ±0.27‰ (1 s.d.).

For total organic carbon (TOC) analyses, finely powdered rock samples from locations shown in Figure S3 were washed in methanol to extract the most relevant contamination, weighed into acid‐cleaned (1% HCl) and combusted (450°C, 5 h) glass vials. The sample materials were subsequently suspended in Milli‐Q‐Water, acidified to pH = 2 with HCl, followed by analysis using a Shimadzu high‐sensitivity TOC‐L_CSH_ analyzer coupled with a suspended‐solids measurement kit. Samples were stirred continuously throughout the analysis to maintain a homogenous suspension. The suspensions were analyzed in triplicate involving the non‐purgeable organic carbon (NPOC) method by combusting at 680°C over a platinum catalyst. TOC concentrations were determined based on a calibration using potassium hydrogen phthalate reference materials (Stubbins and Dittmar 2012). The instrument's limit of quantification, defined as the smallest amount of an analyte that can be reliably quantified by the instrument, was calculated from linear calibration lines following the root mean square method described by Corley (2003), yielding a limit quantification of 9 μg L^−1^. The analytical uncertainty, defined as the standard error from seven repeat measurements of a 100 μg L^−1^ reference sample, was 1.5%. The blank associated with the instrument was determined using the automated blank check program of the TOC analyzer. For this, ‘carbon‐free’ water was generated by passing ultrapure water several times through the catalyst bed at 680°C, collected in an ultrapure water trap and analyzed for organic carbon after re‐injecting it without exposure to the atmosphere. The signal obtained from the analysis of this ‘carbon‐free’ water was used to define the instrument blank, and this value subtracted from the signal during data analysis. The measured organic carbon (OC) concentration of the sediment suspension in μg L^−1^ was converted to weight concentration (wt% OC).

Samples 21‐207, 21‐208, and SR21‐004 were analyzed to obtain δ^13^C values of kerogen. Powdered sample aliquots of 40.1–56.9 g were acid‐digested (HCl and HF) at StratoChem Services in Cairo, Egypt. Following neutralization, a heavy‐liquid separation (200 g/cc ZnCl) was performed, and the suspension was separated from settled materials by decanting. The filtered suspension appeared to be enriched in fine pyrite particulates, whereas the settled material contained undigested silicates and pyrite. For stable carbon isotope analysis, aliquots of ca 0.05–0.4 mg of the pellet were weighed into Sn capsules. The suspension, still on filters, was wiped off with a small piece of pre‐cleaned glass fiber filter and folded into an Sn capsule without gravimetric determination. Following combustion in a Thermo EA‐Isolink, ^13^C was determined with a ThermoFisher Delta V Advantage mass spectrometer at the Deutsche GeoForschungszentrum Potsdam. IAEA CH‐7 (peptone) was used as an internal standard and yielded a long‐term analytical precision of < 0.2‰.

## 3 Results

### 3.1 Stratigraphic Setting of Calcareous Mounds

Moodies Group strata in the Sondeza Range of northernmost Eswatini are dominated by thin‐ to thick‐bedded, planar‐stratified and cross‐bedded, fine‐ to coarse‐grained quartzofeldspathic and lithic, commonly tuffaceous sandstone, interbedded with subordinate siltstone and minor shale and conglomerate (Figure 2). This is a common composition of Moodies strata throughout the central BGB. A volcanic complex reaching a thickness of ca. 450–900 m and consisting of basaltic lava flows, lahars, incised conglomeratic channels, and interbedded volcaniclastic rocks, about mid‐section in the stratigraphy of the Sondeza Range, can be correlated westward along strike (and into the South African part of the BGB) with the Moodies Lava Complex (Anhaeusser 1978; Janse van Rensburg et al. 2021). Strata underlying (to the south of) the volcanic complex are > 2000 m thick and can be subdivided using the standard Moodies Group regional stratigraphy (Anhaeusser 1978). Paleocurrents established from cross‐bedded sandstones are generally directed toward the northeast, consistent with the overall paleocurrent pattern of Moodies Group sandstones (Heubeck and Lowe 1994). We studied closely three sites: Wrinkled and tufted kerogenous laminae interbedded with fine‐ to medium‐grained sandstone at Site 1 within unit MdS1 (Figure S6) are virtually identical in texture and composition to abundant kerogenous laminae preserved in the southern Saddleback Syncline of the central BGB, located ca. 27 km along structural trend to the southwest. Those laminae had been interpreted as fossil shallow‐water benthic microbial mats in coastal‐plain and tidal environments (Noffke et al. 2006; Heubeck 2009; Gamper et al. 2012; Homann et al. 2015). Strata overlying (to the north of) the volcanic complex are tightly folded (Figure 1) and consist of > 300 m thick, fine‐ to coarse‐grained tuffaceous sandstones and thin conglomerates in tidal and coastal facies. They preserve calcareous mounds at Sites 2 and 3 (Figures 1 and 2), described in the following subchapter.

### 3.2 Morphologies of Calcareous Mounds and Laminae

**FIGURE 3:**
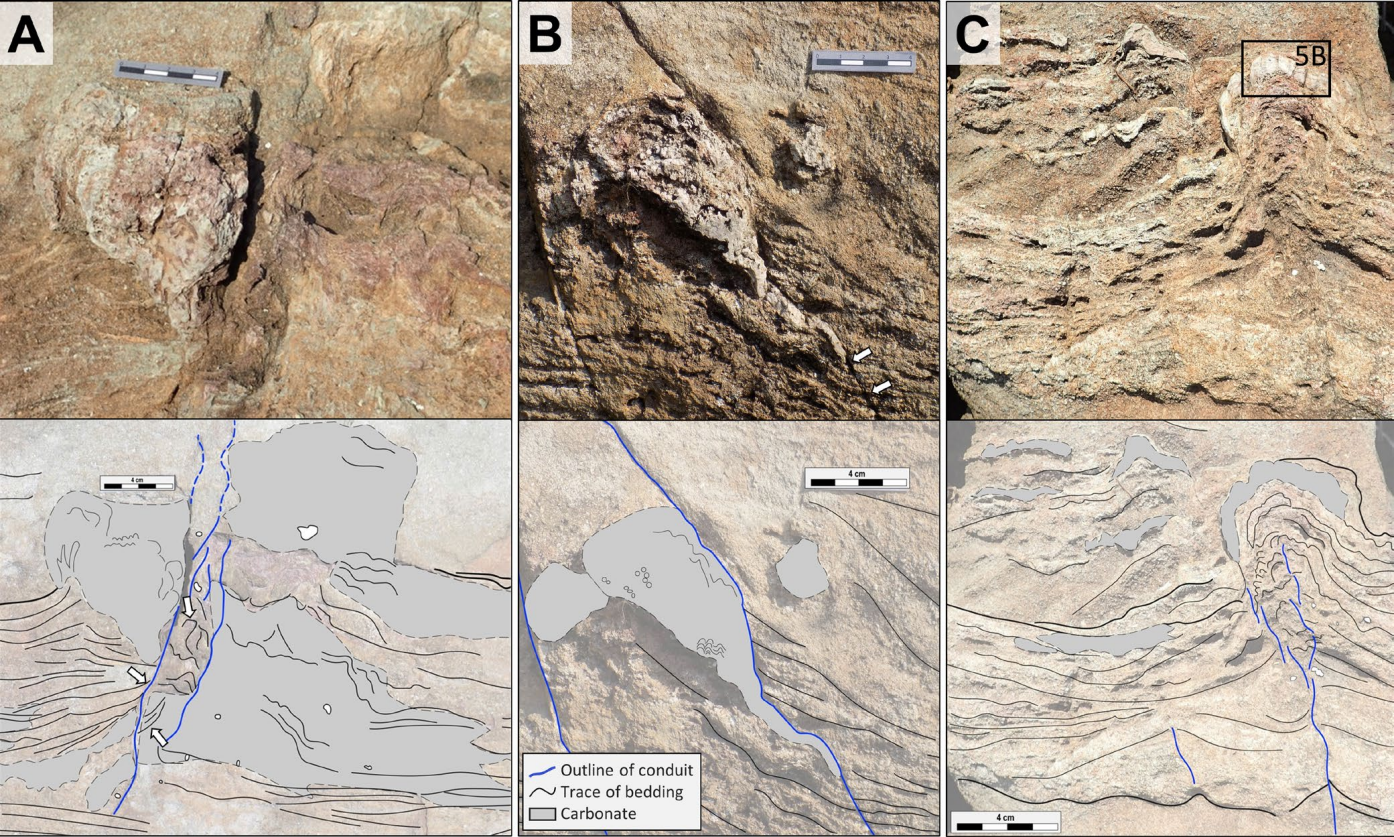
Outcrop photographs (top) and line‐drawings (bottom) of isolated, club‐ to pedestal‐shaped, laminated, partially silicified carbonates that cap fluid‐escape conduits. (A) Inverse conical structure (left) and tapering apron (right) above and to the left of a conduit. Adjacent laminae experienced soft‐sediment deformation (white arrows). (B) Convex‐up calcareous structure overlying a fluid‐escape structure, possibly modified by late minor faulting. (C) Calcareous cap on top of a fluid‐escape structure (sample 19‐117). Sandstone bedding planes are rising towards the conduit.

**FIGURE 4:**
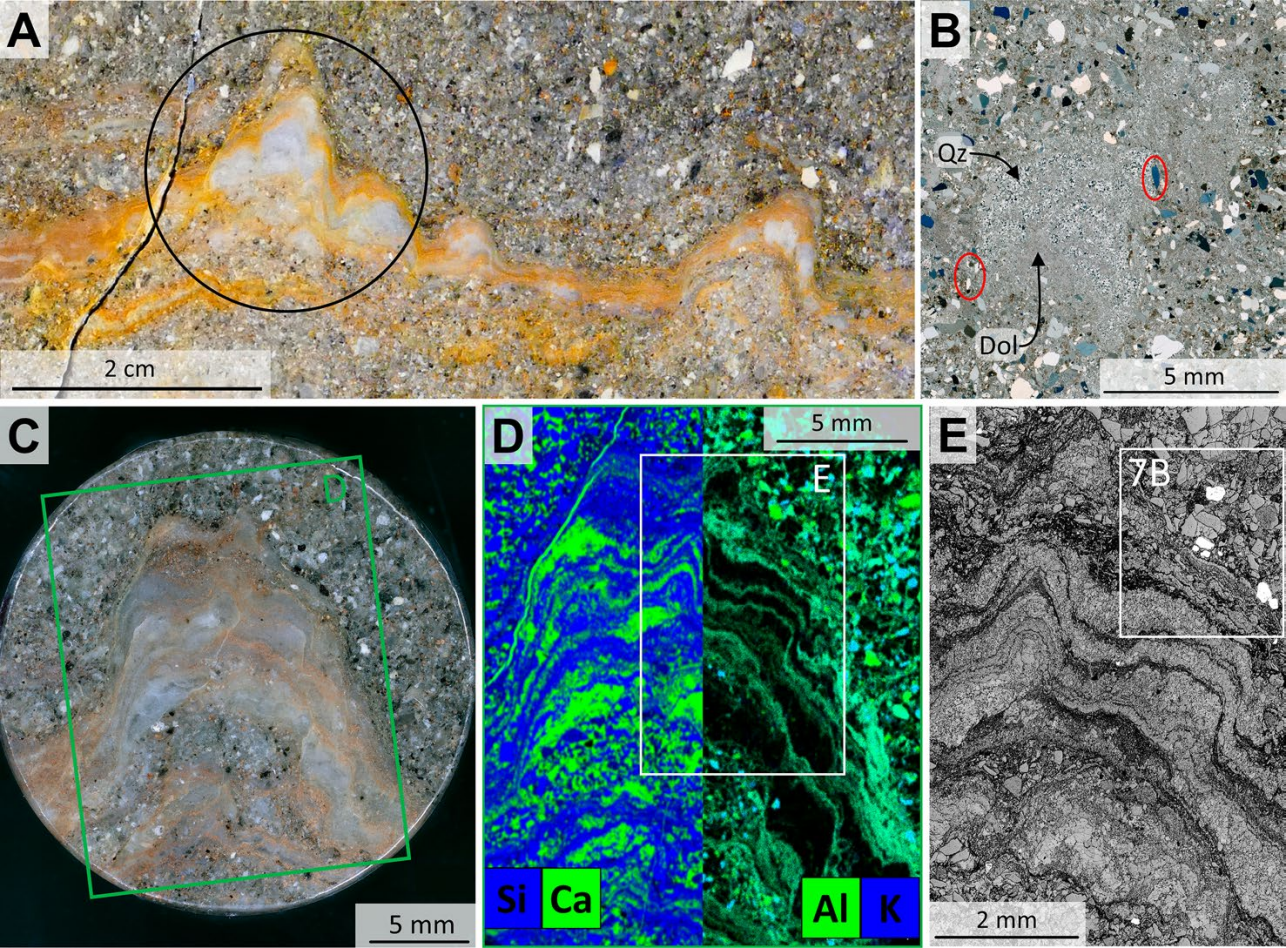
Images from a calcareous, linked, conical mound between undulatory laminae (sample 19‐117, area P1 from Figure S3). (A) Photograph of a polished surface showing two calcareous mounds linked by laminae. Circle indicates the location of the plug from which slices shown in (C) and Figure 8B were taken. (B) Thin‐section microphotograph (crossed nicols) of blocky silicified calcareous mounds free of sericite (sample 19‐117‐γ). The silica occurs in layers and displays the internal lamination which is cemented by dolomite. Red ellipses highlight quartz grains above the angle of repose. (C) Microphotograph of polished surface of hemispherical, stubby mound with steep margins on fine‐grained sandy substrate. Green rectangle delineates the area shown in (D). (D) Composite element map of Si (blue) and Ca (green) to the left and of Al (green) and K (blue) to the right of the mound shown in (C). White rectangle indicates the area shown in (E). (E) High‐resolution grey‐scale microphotograph (reflected light) of area marked by white rectangle shown in (D). Black laminae are enriched in Al, K and Ca (representing carbonate and sericite), light grey laminae are enriched in Si (representing mostly quartz), and white patches represent pyrite.

**FIGURE 5:**
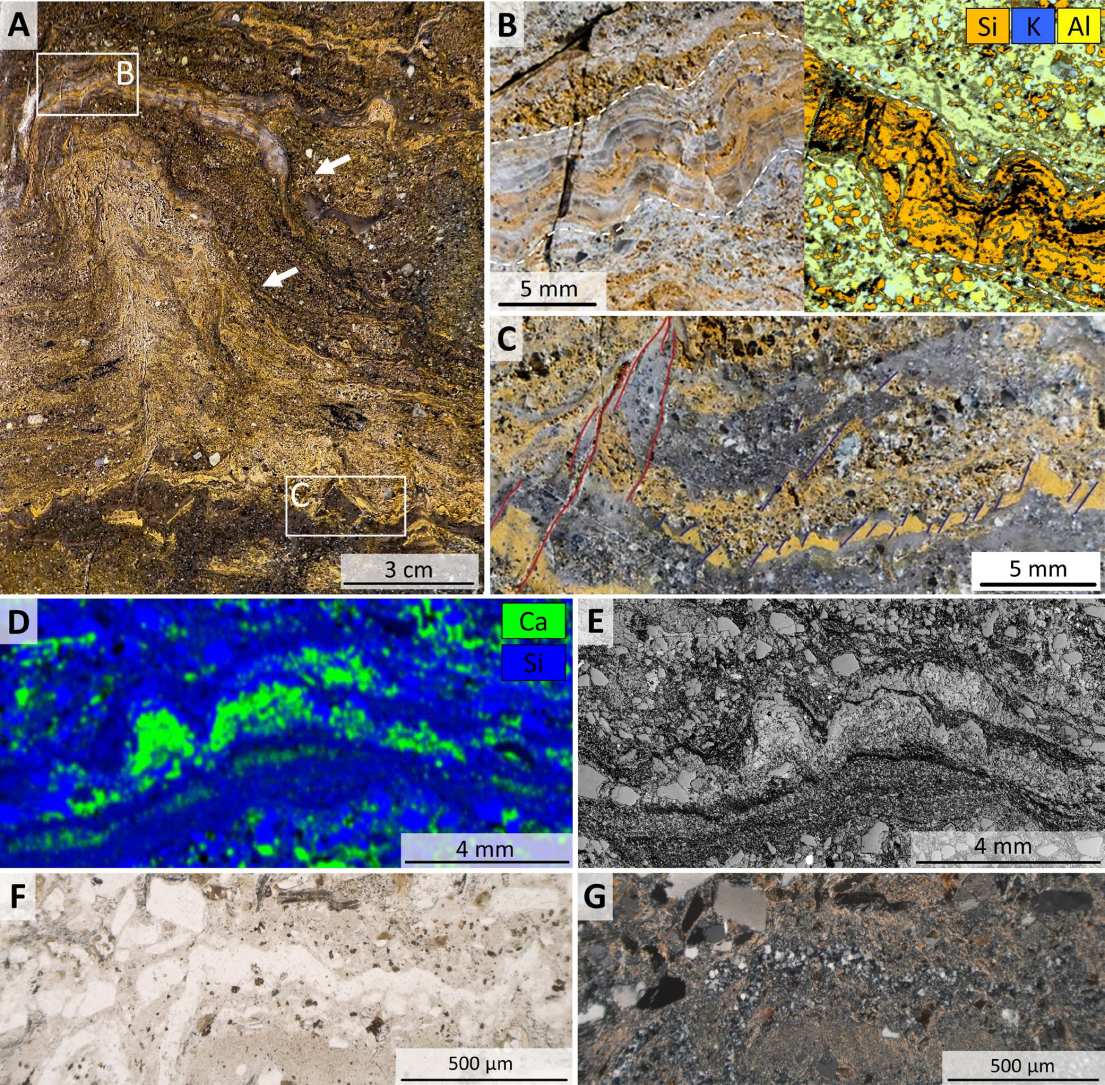
Image sequence from slab‐ to thin‐section scale of a silicified calcareous lamination (sample 19‐117, see also Figure S3). (A) Cross‐section of part of the fluid‐escape structure shown in Figure 3C. The light‐grey calcareous cap pinches out on both sides. Onlap structures occur to the right (white arrows). (B) Close‐up of area shown in A, documenting up to 1 cm thick, stratiform undulatory laminae. Margins are shown by white dashed line. Optical photograph to the left, μ‐XRF elemental map of adjacent area to the right. Laminae are dominantly composed of silica and sericite, the latter following the μm‐scale laminations. (C) Close‐up of area shown in A at the base of the fluid‐escape structure, showing multiple fractures (red and blue lines) separating ochre‐colored tuffaceous shale. The left margins of the mounds are commonly sub‐parallel and dip about 60° (dark blue lines). Shale fragments appear dragged upward near main conduit identified by red lines. (D) μ‐XRF elemental map of a polished plug (sample 19‐117‐P3‐II) showing closely spaced, conical, calcareous laminae. Ca is shown in green. (E) Microphotograph of the same area in reflected light, showing shale beds above and below the mounds. (F) Transmitted‐light photomicrograph showing wavy chert laminae under‐ and overlain by fine‐grained sericitic matrix (sample 19‐117‐5). (G) Microphotograph of the same area (crossed nicols).

We follow the terminology of Grey and Awramik (2020) to describe putative stromatolitic morphologies. Site 3 is a grassy area populated by blocky outcrops, spaced meters to tens of meters apart (Figure 3 and Figure S2). We identified about ten carbonate mounds in outcrop, located within thin‐ to medium‐bedded, fine‐ to coarse‐grained feldspathic litharenites and lithic arkoses. The calcareous mounds appear offwhite with a high relief on weathered surfaces (Figure 3; Figures S1 and S2). In well‐exposed outcrops and on cut‐and‐polished surfaces, they exhibit densely spaced, undulatory, columnar to pseudocolumnar lamination defined by variable proportions of carbonate, quartz, and sericite (Figure 4D). Laminae thickness ranges from sub‐mm to ca. 2.5 cm. Petrographic thin sections and Raman spectroscopy confirm dolomite and siderite as the dominating carbonate phases (Figure S9); minor calcite fills fractures (Figure 4D). Sandstone matrix and mounds are partially silicified, which is common in Moodies Group strata throughout the BGB. μ‐XRF scans of carbonate laminae show high contents of calcium, magnesium, and iron (Figures 4D and 5B,D; Figures S4 and S5).

The three largest laminated carbonate mounds define an end member of a range of structures. They occur as isolated, club‐ to pedestal‐shaped structures, reach 9 to 15 cm in height and 5 to 8 cm in width (Figure 3 and Figure S1) and are associated with concave‐upward‐bent bedding planes in the underlying sandstones. The central conduits between the upward‐curved panels show disrupted bedding and incorporate fragments of underlying fine‐grained sandstones and of tuffaceous clasts up to 1 cm in diameter (Figure 3A,C). In some cases, conduits appear to bypass (Figure 3A,B) the calcareous mounds or terminate below them (Figure 3C). Thin, fine‐ to coarse‐grained sandstone beds mm to cm in thickness onlap the flanks of the structures (Figures 3A, 5A, and 9A,B faulting along thin, subvertically oriented discontinuous brittle structures is minor (mm to few cm); those structures do not extend beyond the near environment of the carbonate mounds (Figures 5B and 9A). Each structure can be best interpreted as a fluid‐escape structure in poorly consolidated, water‐saturated sand which underwent minor and early brittle adjustment by being loaded by a topping carbonate mound (van Loon 2009).

**FIGURE 6:**
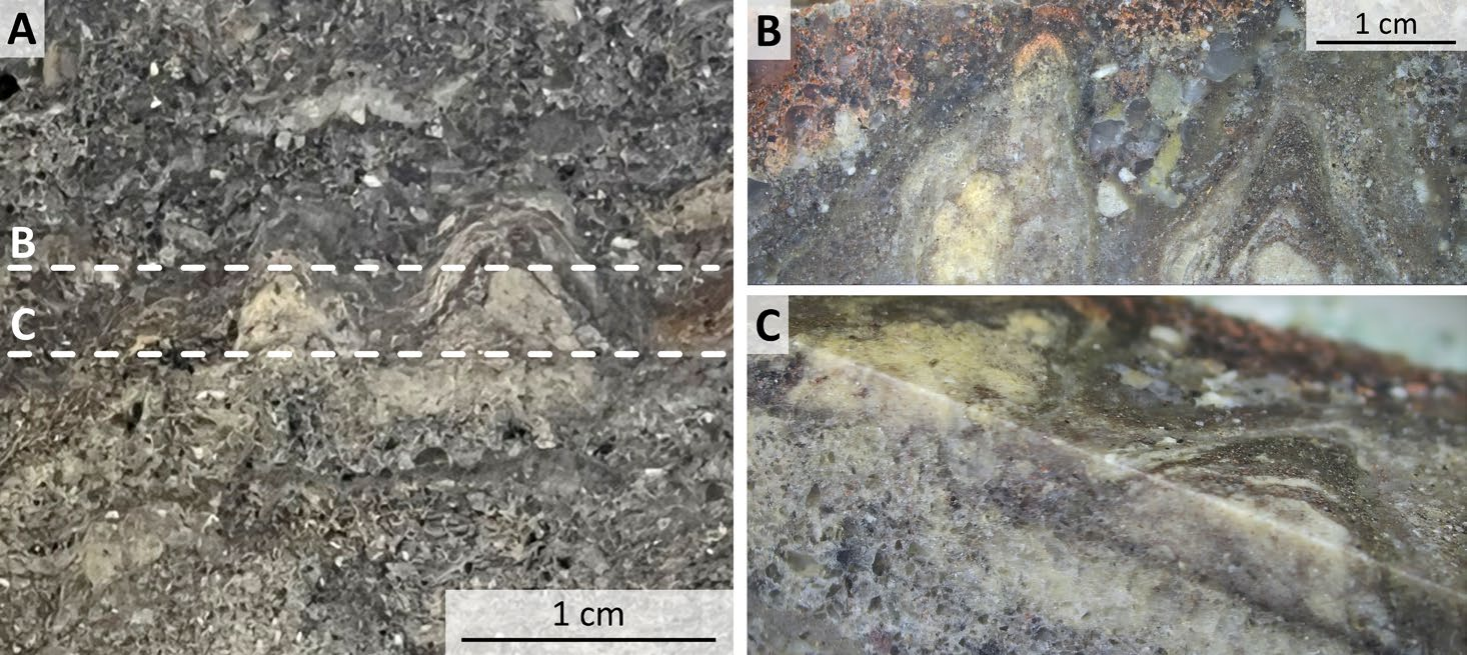
Three‐dimensionality visualized by serial horizontal slabbing of (A) two small, domal, linked mounds with flat base within thinly bedded sandstone (sample 21‐214). White dashed lines display locations of the horizontal cuts shown in B and C. (B) The mounds show conical to wedged shapes with a distinct internal lamination and terminate within a few cm. (C) Oblique view showing the consistent geometrical relationships between vertical and horizontal transects. Horizontal black line is subparallel to bedding.

**FIGURE 7:**
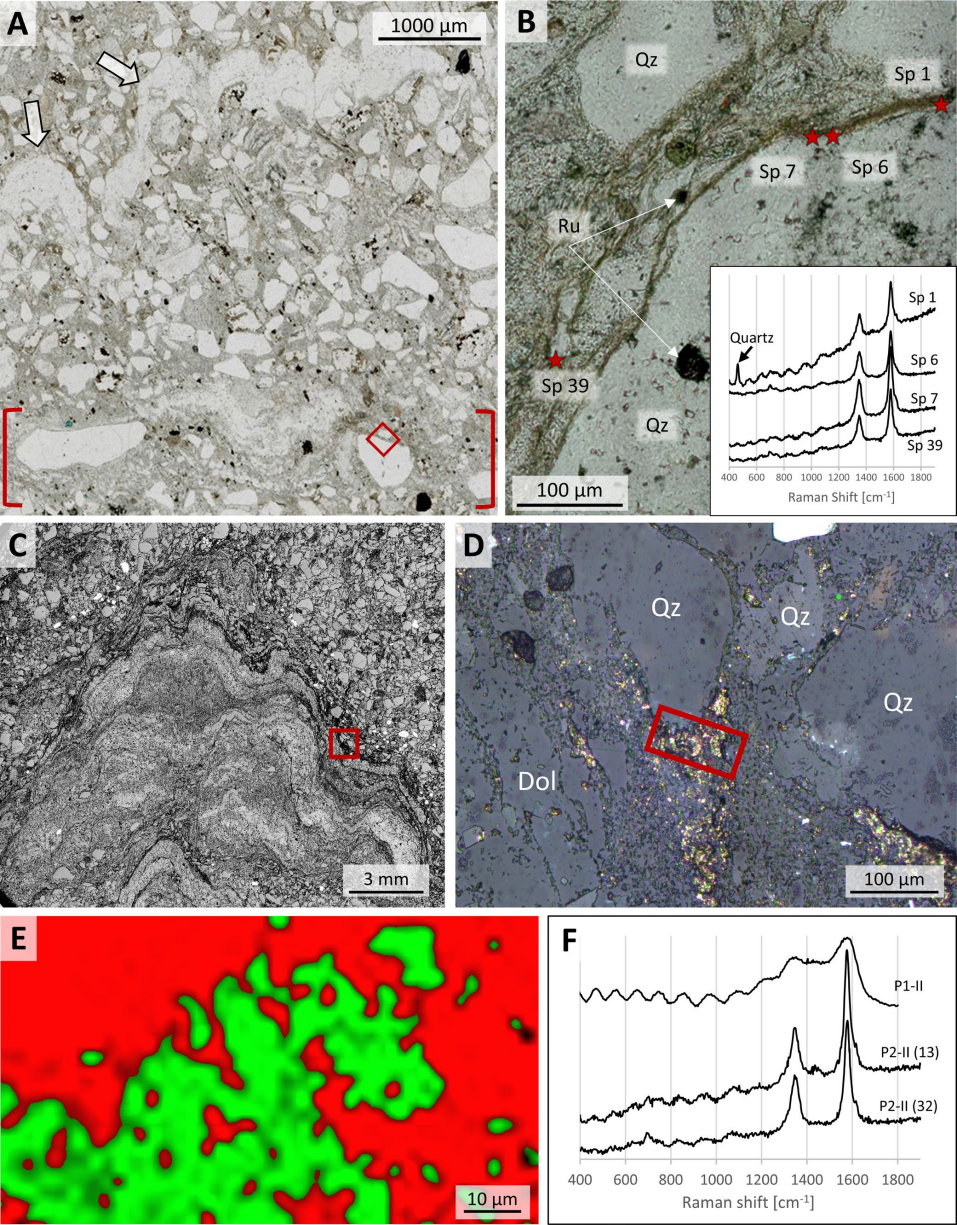
Remnants of carbonaceous material at Site 3 (sample 21‐214‐II). (A) Photomicrograph under plain light of fine‐ to medium‐grained sandstone with a domical‐shaped micro‐quartz band (white arrows) and a calcareous sericite bed (red brackets). (B) Photomicrograph of the area shown in A (red square) showing the intergrowth of quartz (Qz), rutile (Ru), and dark brown carbonaceous matter. The location and uncorrected spectra (inset) of four representative Raman measurements (red stars) highlight and characterize the distribution of carbonaceous matter. (C) Monochrome high‐resolution microphotograph of sample 19‐117‐P1‐II, using coaxial reflected light under a Keyence digital microscope. Black laminae at the margins of the mound incorporate measurable kerogen. A clear margin of the carbonate mound outlined by the red rectangle is shown in (D). (D) Reflected‐light map of the area shown in C (red rectangle). Grains to the right represent quartz (Qz) and, to the left, dolomite (Dol), respectively. The black laminae shown in C are dominated by a micritic sericite‐quartz composition. The areas with a high (“golden”) reflectance overlap with a kerogen signal. (E) Raman map of area shown in D (red rectangle) showing the distribution of kerogen (green) to other mineral phases (red) and spots with no data (black). (F) Uncorrected Raman spectrum for kerogen shown in E and representative spectra from sample 19‐117‐P2‐II. The uppermost graph shows an altered kerogen signal with distinguished D and G peaks.

The opposite end member consists of mm‐ to μm‐scale, undulatory layered laminae defined by alternating micro‐quartz, sericite, and carbonate. These laminae occur a few decimeters from and adjacent to the fluid‐escape structures described above (Figures 5F,G and 7A; Figure S8) in horizontally stratified, fine‐grained sandstones which are commonly interbedded with siltstone and shale. They link meso‐scale, stubby, steep‐margined, hemispherical or conical carbonate mounds up to 30 mm in height and 60 mm in width (Figures 4, 6, and 10; Figures S4, S7, and S10) and may grade into calcareous, conical, domed, and tufted structures (Figure 5D; Figure S8) which show increasing thickness up to ~10 mm above small fluid‐escape conduits (Figure 5A,B). Three‐dimensional exposures and serial cuts of plugs and slabs (Figures 6 and 10; Figure S10) show that these meso‐scale mounds may form ridges of > 10 cm length and possess intricate internal layering (Figures 6 and 10). Only in a few cases was serial slabbing successful (Figure 10) as mounds often terminated within a few mm (Figure 6B) or changed direction horizontally (Figure S5A).

**FIGURE 8:**
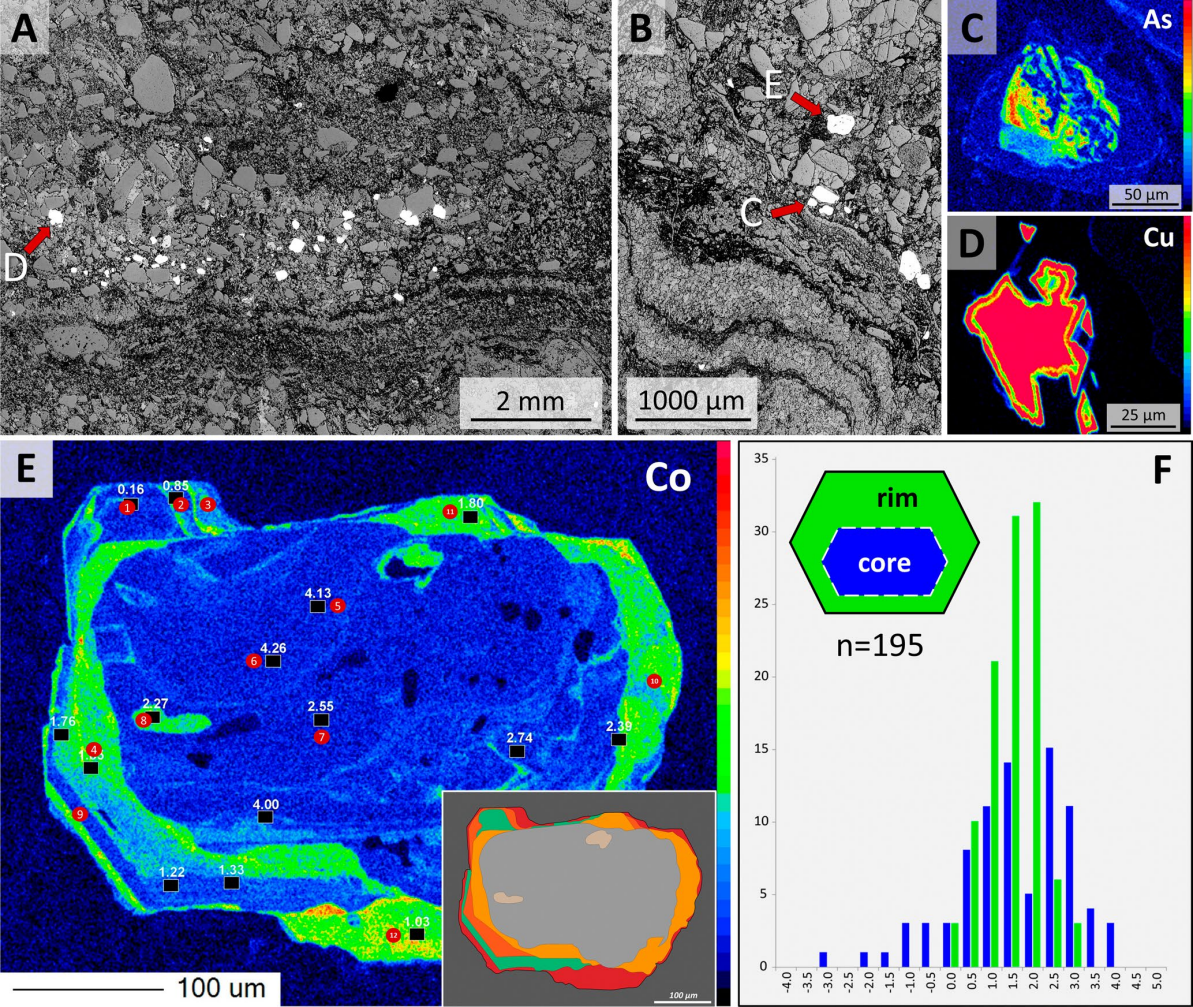
Analyses of detrital sulfide. (A, B) Monochrome microphotographs of euhedral pyrite grains (white) deposited just above a calcareous lamina. (A) Sample 19‐117‐P3‐IV, (B) 19‐117‐P1‐I, both in reflected light. Red arrows indicate the locations of grains shown in Figure 8C–E. (C, D, E) Element distribution maps of sulfide grains; color scale bars reflect element concentration (red = high; dark blue = low). (C) Arsenic concentration in a porous core of a representative grain. (D) Cu concentration in an anhedral chalcopyrite grain. (E) Co concentration in a zoned euhedral pyrite grain. Inset shows four generations of authigenic overgrowth around the porous detrital core. Filled red circles indicate locations of measurements of total elemental composition (Table S1). Black squares represent locations measured for δ^34^S_VCDT_; these values are stated in white. (F) Histogram of SIMS δ^34^S analyses, comparing values from the authigenic rims (green) with those from the detrital core (blue). δ^34^S values do not differ substantially, lending little support to an interpretation of biomediated S processing.

Laminae within the mounds contain isolated detrital grains oriented subparallel to bedding (Figures 4B and 7C; Figure S8). Thin‐section petrography and polished hand‐sample slabs show that detrital pyrite, rutile, and quartz grains preferably accumulated along the margins of and within the mounds (Figures 4B and 8B). Some mounds show poorly defined margins, lacking internal lamination (Figure 5D,E and Figure S5A). Calcareous laminae also occur as cm‐long fragments or rip‐up clasts within the basal fluid‐escape conduits, but also within overlying sediment (Figure 5A,C). Segmented, ochre‐colored, fine‐grained sediment beds occur at the base of larger fluid‐escape structures (Figure 5C).

### 3.3 Raman‐Spectroscopic Determination of Kerogen and Peak Temperature

Kerogenous material occurs as up to 10 μm thick, continuous and undulating laminae, best observed in thin sections (Figure 7A,B and Figure S6B–E), and as finely dispersed areas up to 60 μm in diameter (Figure 7E) within the calcareous mounds, with high reflectivity and fluorescent Raman spectra (Figure 7F). Raman spectroscopic analysis of carbonaceous matter (RSCM) from seven samples yielded unambiguous kerogen spectra (Figure 7F and Figure S6F). Characteristic D and G peaks are moderately to well defined in carbonaceous matter from laminae and moderate in carbonaceous matter from calcareous mounds. 56 measurements yielded spectra that were of sufficient quality to determine maximum peak temperature (Table S2). Measurements from Site 3 yielded values of ~430°C to ~530°C with a mean of ca. 500°C, significantly higher than samples from Site 1 (Table S2) and also higher than values recorded from Moodies carbonaceous matter in the central BGB (Stengel et al. 2024).

### 3.4 Carbon Isotope Measurements

Analysis of TOC from three silty shale samples of specimen 19‐117, incorporating dark laminae and taken at Site 3 near a fluid‐escape structure (for location see Figure S3), yielded values between 0.0008 and 0.0064 wt‐%, placing them far below the amount required (approx. 0.01 wt%) for laboratory determination of organic‐carbon δ^13^C isotopic values. Analysis of three wrinkled‐laminated sandstone samples from Site 1 (Figure S6), incorporating cm‐thick beds of condensed dark kerogenous laminae interbedded with shaly siltstone, was conducted. After processing, the filtrate from two of three samples yielded concentrations of organic carbon of 25.2 wt‐% with δ^13^C values of −27.3‰ (sample SR21‐004) and 5.0 wt‐% with −26.2‰ (sample 21‐207; Figure S1), respectively.

**FIGURE 9:**
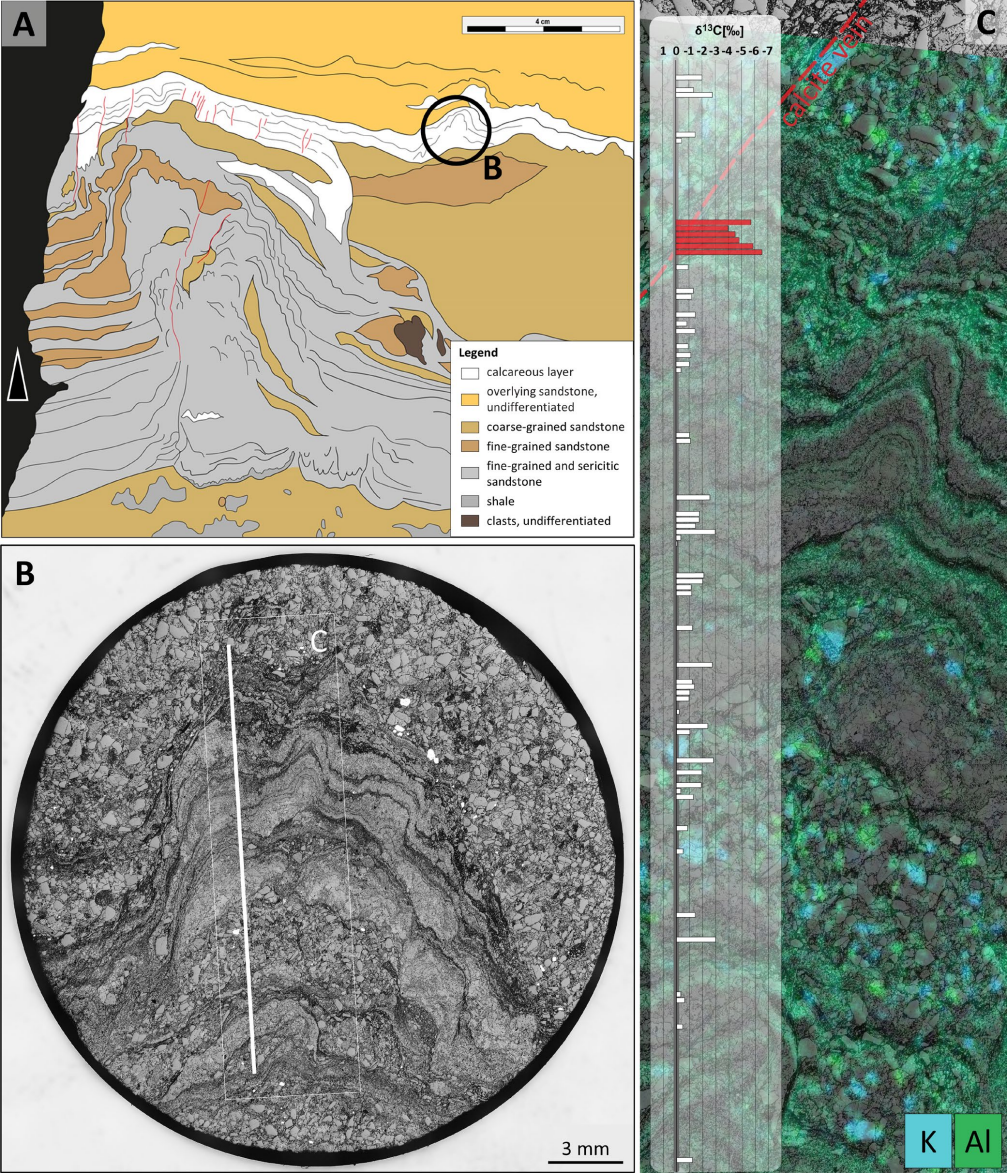
(A) Sketch of the same fluid‐escape structure as shown in Figures 3C and 5A but from a different point of view. To highlight differences, color‐coded areas are homogenized according to their dominant mineral composition. Red lines illustrate vertical fractures. The black circle illustrates the position of the calcareous mound used for δ^13^C_carb_ isotopic analysis and shown in Figure 9B. (B) Polished plug (19‐117‐P1‐I; coaxial reflected light) highlighting internal lamination. Isotope measurements were taken along a traverse (white line) perpendicular to the lamination. (C) μ‐XRF elemental map of area delineated by white rectangle in Figure 8B, overlain by bar plot of δ^13^C_carb_ values along the white line shown in Figure 8B. δ^13^C_carb_ values range between 0‰ and −4‰, except for values decreasing to −7‰ within and near a calcite vein (red bars). Al (green) and K (blue) largely represent sericite.

In‐situ SIMS isotope determinations of δ^13^C_PDB_ from sample 19‐117‐P1‐I (*n* = 93) along a linear traverse perpendicular to laminae of a stubby hemispherical carbonate mound yielded values ranging between −2.5‰ and 0.5‰ but lacked a recognizable trend (Figure 9C). A small population of outliers along a calcite‐filled fracture yielded values ranging from −4.0‰ to −6.6‰. These results do not differ significantly from measurements of δ^13^C_carb_ cement from over‐ and underlying sandstones.

### 3.5 Geochemistry and Composition of Pyrite and Heavy Minerals

Pyrite grains, often aligned in minute placer deposits, vary in diameter from 35 to 440 μm; they show anhedral and euhedral shapes (Figure 8A,B,E). Microprobe total element measurements and mapping show low, homogeneously distributed concentrations of Mo, Pb, Sb, Ni, and Au, but zoned growth of Co, As, and Cu concentrations (Figure 8C–E), including up to four generations of overgrowth, defined by strong variation in Co concentration (Figure 8E). Arsenic occurs in higher concentration in complexly zoned cores of four grains (Figure 8C). Cu occurs sporadically at very high concentration as inclusions in porous pyrite cores and as detrital chalcopyrite grains up to 50 μm in diameter (Figure 8D). Pb and As occur as inclusions of galena and arsenopyrite in porous cores. δ^34^S_VCDT_ values of authigenic rims and detrital cores of pyrite vary unsystematically between 3.5‰ and 4.0‰ (e.g., Figure 8F and Table S1).

## 4 Discussion

### 4.1 Mode and Timing of Carbonate Formation

Carbonates may form at Earth's surface by biogenic or abiogenic processes. Distinguishing between these in ancient sedimentary strata affected by diagenesis, metamorphism, and deformation is complex and can be contentious. The spatial and temporal relationship of fluid‐escape structures to capping carbonates and mounds raises the question of whether and how these structures are genetically related. The partially silicified carbonate caps (Figures 3C, 5A, and 9A) are rarely traversed by fluid‐escape conduits; caps therefore were either mechanically sufficiently strong to divert fluid flow around them or postdated the last major phase of fluid expulsion. Some calcareous mounds (Figure 3B) appear to plug the fluid‐escape structures not unlike a bottle cork, and others appear to show a complex multi‐stage history of vertical dewatering, upward sediment transport, carbonate growth, cementation, soft‐sediment deformation, and locally minor brittle displacement (Figure 3A). The meso‐ to micro‐scale mounds and laminae (Figures 4, 5, S4C and S7), in contrast, in places show evidence of segmentation, rotation, and displacement within unconsolidated granular sediment but generally do not show brittle deformation suggestive of brecciation due to structural deformation or tectonics. In all cases, however, the onlap of sandstone bedding planes indicates that the growth of the mounds and the associated development of paleotopography or ‐bathymetry predated adjacent sand deposition (Figure S7C,E).

### 4.2 Abiogenic Origin of Carbonate Mounds and Laminae?

The intricate association of carbonates with the fluid‐escape structures and the increasing thickness of the calcareous beds and laminae towards them suggest a close temporal interaction between mound growth, expelled fluids, and carbonate precipitation.

1. Regional heating of pore waters by the Moodies Lava complex nearby may have caused excessive pore pressure of fluids saturated in CO_2_, which was by springs along the fluid‐escape structures. Pressure decrease and CO_2_ degassing caused carbonate precipitation (e.g., Des Marais and Walter 2019), comparable to the formation mode of travertine (Brasier 2011).
2. Soft‐sediment deformation or ductile tectonics may produce structures resembling the investigated structures (cp. the controversial discussion of potential stromatolites or ductile mullions from the Isua Supracrustal Belt (Nutman et al. 2016; Zawaski et al. 2020; van Kranendonk et al. 2025)). Vaguely similar flame structures (Figure S8) and deformed quartz bands (Figure 5D–G; e.g., van Loon 2009; Suter et al. 2011) locally occur in the metasedimentary rocks of the study area.
3. Our geochemical analyses do not explicitly support a biogenic origin of the carbonate mounds: The concentration of organic matter in and near the carbonate beds is very low. The high δ^13^C_carb_ values (Figure 9C) are broadly consistent with inorganic precipitation (Ohmoto et al. 1993; Schidlowski 2001; Shen et al. 2001; Roerdink et al. 2012; Martin et al. 2013). A large number of pyrite grains from the margins of the calcareous mounds show a narrow δ^34^S distribution near 0‰, lending little support to biogenically‐mediated diagenetic precipitation (Thode 1991), which would be expected to range between approx. −5‰ and −40‰ (Shen et al. 2001).

### 4.3 Biogenic Origin of Carbonate Mounds and Laminae?

The arguments presented in the previous section in favor of an abiogenic origin of the mounds can also be approached from a different perspective. First, abiotic travertine precipitates are generally laterally continuous and show smooth depositional patterns without significant thickness variations over short distances (Lowe 1994; Rainey and Jones 2009), unlike the structures discussed here. Secondly, the geochemical data which fail to support biogenic fractionation of C and S isotopes may not be fully valid because the presence of abundant sericitic grains in the host rock sandstone indicates that significant hydrothermal alteration affected feldspar and clay, the elevated ^13^C values in a calcite‐filled fracture shown in Figure 9C demonstrate that isotope systems were disturbed, and the high metamorphic peak temperature may indicate that kerogen was nearly eradicated, explaining the low TOC. Combinations of similar data patterns have been observed, albeit usually to a lesser degree, in other sedimentary rocks of the BGB (Tice et al. 2004; Hofmann 2005; Bao et al. 2007; Heubeck et al. 2022).

Carbonate mounds and laminae in the study area thus could have formed biogenically in several ways, suggested by the presence of kerogen, their association with structures interpreted elsewhere as microbial mats, a delicate μm‐scale internal lamination, and the morphological similarity to modern stromatolitic structures (e.g., Ionescu et al. 2015). We discuss these arguments below.

**FIGURE 10:**
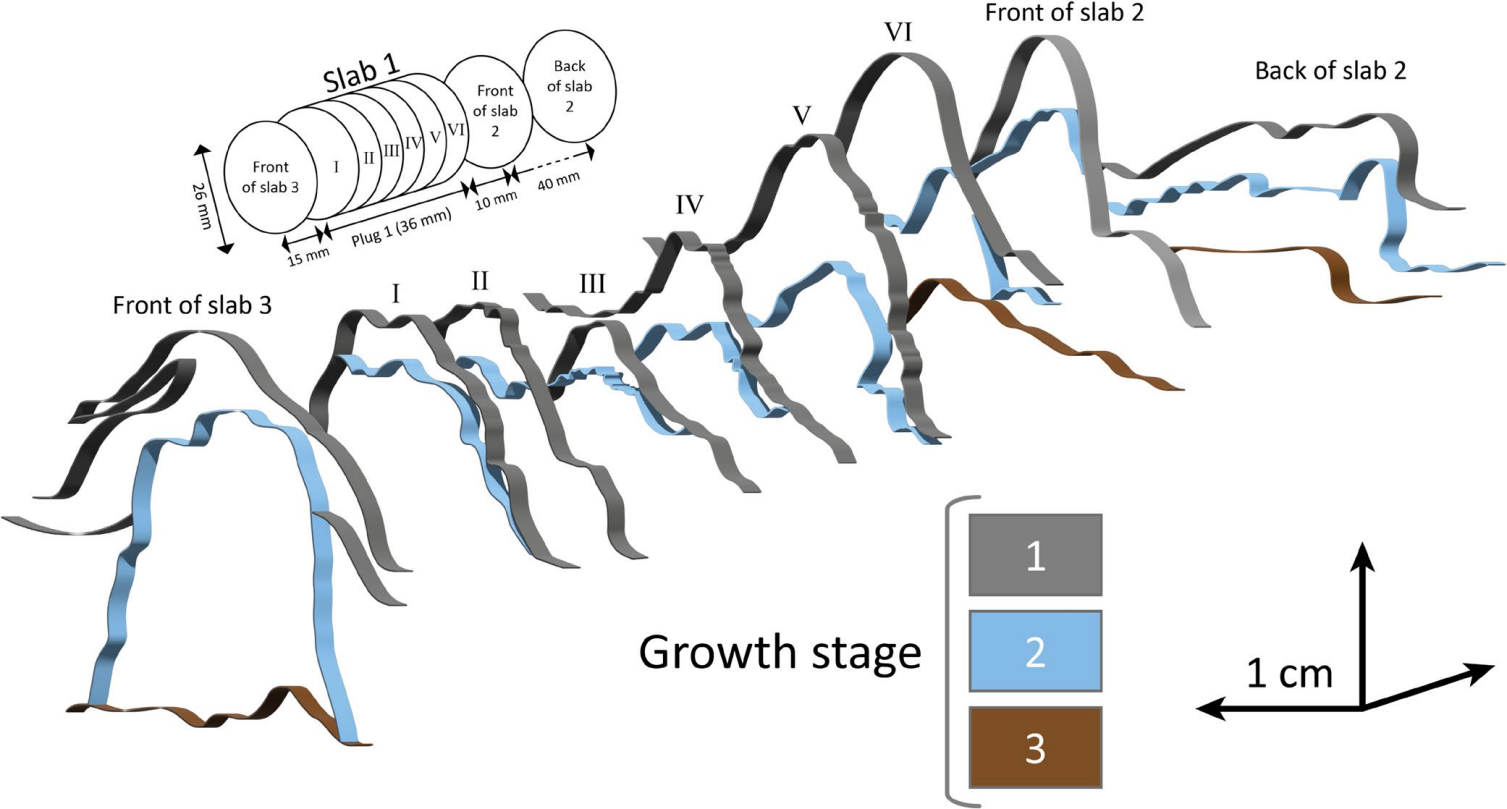
3‐D model of a representative mound from sample 19‐117‐P1 based on serial cuts of Plug 1 and surrounding slabs, showing three growth phases within a length of ~10 cm. Slice P1‐I is also shown in Figure 9B, displaying delicate internal lamination.

1. Homann et al. (2015, 2016) documented early‐diagenetic carbonate precipitation in elongate cavities below microbial kerogenous laminae in correlative units of the Moodies Group in South Africa. They interpreted them as having formed due to a biogenic redox process. Thus, a carbonate–microbial relationship in the Moodies Group is well established. Although microbial laminae (at Site 1) and carbonate mounds (at Sites 2 and 3) are found stratigraphically separated, they share the same region, tidal facies favorable to microbial life, carbonate composition, and metamorphic grade.
2. The morphology of the calcareous mounds, including the highly variable thickness and upward‐thickening of laminae, the domical to conical shapes (Figures 6 and 10; Figure S10), the linked columnar to pseudo‐columnar architecture, the steep‐sided walls resulting in the onlap of sediment laminae (Figures 3A and 4C), the discordance of laminae within the structure (Figure 9B), and the termination of carbonate precipitation by clastic sediment avalanches (van Kranendonk 2007, 2011; Figures 4D and 5B; Figure S4D) all resemble the morphology of ancient as well as recent meso‐ to microstromatolites (e.g., Bosak et al. 2012; Duda et al. 2016; Grey and Awramik 2020; Vahrenkamp et al. 2024).
3. Detrital grains oriented subparallel to the layering of mounds and laminae (Figure 4B and Figure S8B) above the angle of repose may have adhered to the steep slopes of domes due to sticky extra‐cellular polymeric substance (EPS; Flemming and Wingender 2001a; Wacey 2009; Noffke 2010). EPS is a secretion that facilitates attachment of structured ‘biofilm’ communities to surfaces (Flemming and Wingender 2001b; Flemming et al. 2007; Decho and Gutierrez 2017). The extent of its functionality is a subject of debate. Proposed roles include the mediation of carbonate formation (e.g., Al Disi et al. 2019), the potential utilization as nucleation sites conducive to the early stages of crystal growth (e.g., Arp et al. 1999; Ionescu et al. 2015; Flemming et al. 2023), and the modification of the microenvironment through incorporation of negatively charged functional groups (e.g., Neu 1996).
4. TOC‐L_CSH_ analyses may, and Raman spectroscopy do confirm the presence of kerogen within the wrinkled laminae and the sericitic laminae of the calcareous mounds (Figure 7). The sericitic, kerogenous, and calcareous textures are closely spaced and grade into each other, which supports their cogenetic formation (Figure 4D). The mineralization of calcareous structures is presumably controlled by microbial EPS. δ^13^C values of samples from Site 1 are consistent with those of photosynthetic organisms (Schidlowski 2001).

The formation within a habitable tidal environment, the occurrence of regionally correlated kerogenous microbialites at Site 1, the stromatolitic morphology of the calcareous mounds, the indirect evidence of the former presence of EPS, and the occurrence of kerogen within carbonate laminae and mounds collectively support a biogenic origin of the described structures. It would be tempting to speculate on the sources and composition of fluids and their potential utilization in the metabolisms of diverse microorganisms, possibly even acting as consortia. Arguments discussing the role of H_2_, CH_4_, CO_2_, or H_2_S, however, can, at present, only be supported by recent analogs and by the plausible regional geologic context, but not by analytical data. Metabolic processes are thus currently unidentifiable in these very old, complex, and altered strata.

## 5 Conclusions

Decimeter‐ to millimeter‐sized, calcareous, partially silicified mounds and laminae are commonly associated with fluid‐escape structures in a siliciclastic tidal setting of Moodies Group strata (ca. 3.22 Ga) of the Barberton Greenstone Belt in Eswatini. Some of these structures meet all criteria for their classification as stromatolites. Geochemical data are inconclusive, owing to significant thermal and metamorphic alteration. If the carbonate mounds were indeed at least partially biogenic, their presence on top of and within fluid‐escape conduits would suggest that reactants dissolved in emanating fluids played a role in supporting metabolisms which precipitated carbonate.

## Conflicts of Interest

The authors declare no conflicts of interest.

## Supporting information


**Table S1:** Microprobe total element measurements of pyrite of sample 19‐117‐P1‐I, grain I.

**Table S2:** Paleotemperature calculations.

**Figure S1:** Outcrop documentation of calcareous structures at Site 2.
**Figure S2:** Outcrop documentation of calcareous structures at Site 3.
**Figure S3:** Description of sample 19‐117 — locations of plugs and thin sections.
**Figure S4:** Description of sample 21‐214 — cm‐scale silicified and calcareous mounds.
**Figure S5:** Morphological variation and composition of sample 19‐117‐P4.
**Figure S6:** Description of wrinkly laminae at Site 1.
**Figure S7:** Variation of cm‐scale silicified and calcareous stromatolites (sample 19–117).
**Figure S8:** Possible small‐scale soft‐sediment deformation (sample 19–117‐α).
**Figure S9:** Representative Raman spectra of Quartz, Dolomite, and Siderite.
**Figure S10:** Three‐dimensionality of mini‐mounds visualized by serial slabbing of slab of sample 21–214.

## Data Availability

Data supporting the findings of this study are available in the Supporting Information of this article.
