# Supplementary material for: Stromatolitic Mounds in Tidal‐Facies Sandstones of the Paleoarchean Moodies Group (Barberton Greenstone Belt, Eswatini)
Source: Geobiology. 2025 May 2;23(3):e70020. doi: 10.1111/gbi.70020 (PMC12047067; doi:10.1111/gbi.70020)
Supplement: Supplementary file 1 — Table S1: Microprobe total element measurements of pyrite of sample 19‐117‐P1‐I, grain I. [file GBI-23-e70020-s002.docx]

**S table 1 - Microprobe total element measurements of pyrite of sample 19‐117‐P1‐I, grain I.**

| **Point** | **Sample number** | **As (Mass%)** | **Ni (Mass%)** | **Fe (Mass%)** | **S (Mass%)** | **Au (Mass%)** | **Cu Mass%)** | **Co (Mass%)** | **Total (Mass%)** |
| --- | --- | --- | --- | --- | --- | --- | --- | --- | --- |
| 1 | P1-I-01-1 | b.d.l. | 0.1110 | 46.2290 | 55.6220 | b.d.l. | 0.0020 | b.d.l. | 101.9640 |
| 2 | P1-I-01-2 | b.d.l. | 0.0070 | 46.3180 | 55.0830 | 0.0510 | 0.0010 | b.d.l. | 101.4600 |
| 3 | P1-I-01-3 | b.d.l. | 0.0110 | 45.5140 | 54.2570 | 0.0210 | 0.0120 | 1.0390 | 100.8540 |
| 4 | P1-I-01-4 | 0.0290 | 0.0290 | 45.3350 | 53.2360 | 0.0040 | 0.0050 | 0.7570 | 99.3950 |
| 5 | P1-I-01-5 | b.d.l. | 0.0270 | 46.5390 | 54.2150 | b.d.l. | b.d.l. | 0.0070 | 100.7880 |
| 6 | P1-I-01-6 | b.d.l. | 0.0910 | 46.3950 | 54.3810 | 0.0710 | 0.0120 | b.d.l. | 100.9500 |
| 7 | P1-I-01-7 | b.d.l. | 0.0950 | 46.7930 | 54.4210 | b.d.l. | b.d.l. | b.d.l. | 101.3090 |
| 8 | P1-I-01-8 | 0.5520 | b.d.l. | 45.3330 | 55.3600 | b.d.l. | b.d.l. | 1.0060 | 102.2510 |
| 9 | P1-I-01-9 | b.d.l. | b.d.l. | 45.6180 | 54.2560 | b.d.l. | b.d.l. | 0.8650 | 100.7390 |
| 10 | P1-I-01-10 | 0.0170 | 0.0350 | 46.1150 | 54.3160 | 0.0110 | b.d.l. | 0.4730 | 100.9670 |
| 11 | P1-I-01-11 | 0.3150 | 0.0210 | 45.9860 | 54.0400 | 0.0460 | b.d.l. | 0.9880 | 101.3960 |
| 12 | P1-I-01-12 | b.d.l. | 0.0020 | 45.2210 | 54.4440 | 0.0160 | 0.0280 | 1.5660 | 101.2770 |
| 13 | P1-I-01-13 | b.d.l. | b.d.l. | 46.8240 | 54.4450 | 0.0180 | b.d.l. | b.d.l. | 101.2870 |
| 14 | P1-I-01-14 | b.d.l. | 0.0190 | 45.5020 | 54.3960 | 0.0580 | 0.0110 | 1.1790 | 101.1650 |
| 15 | P1-I-01-15 | 5.2340 | b.d.l. | 45.5320 | 50.7720 | b.d.l. | 0.0330 | b.d.l. | 101.5710 |
|  |  |  |  |  |  |  |  |  |  |
| **Point** | **Sample number** | **As (Norm%)** | **Ni (Norm%)** | **Fe (Norm%)** | **S (Norm%)** | **Au (Norm%)** | **Cu (Norm%)** | **Co (Norm%)** | **Total (Norm%)** |
| 1 | P1-I-01-1 | b.d.l. | 0.1090 | 45.3390 | 54.5510 | b.d.l. | 0.0020 | b.d.l. | 100.0000 |
| 2 | P1-I-01-2 | b.d.l. | 0.0070 | 45.6510 | 54.2900 | 0.0500 | 0.0010 | b.d.l. | 100.0000 |
| 3 | P1-I-01-3 | b.d.l. | 0.0110 | 45.1290 | 53.7980 | 0.0210 | 0.0120 | 1.0300 | 100.0000 |
| 4 | P1-I-01-4 | 0.0290 | 0.0290 | 45.6110 | 53.5600 | 0.0040 | 0.0050 | 0.7620 | 100.0000 |
| 5 | P1-I-01-5 | b.d.l. | 0.0270 | 46.1750 | 53.7910 | b.d.l. | b.d.l. | 0.0070 | 100.0000 |
| 6 | P1-I-01-6 | b.d.l. | 0.0900 | 45.9580 | 53.8690 | 0.0700 | 0.0120 | b.d.l. | 100.0000 |
| 7 | P1-I-01-7 | b.d.l. | 0.0940 | 46.1880 | 53.7180 | b.d.l. | b.d.l. | b.d.l. | 100.0000 |
| 8 | P1-I-01-8 | 0.5400 | 0.0000 | 44.3350 | 54.1410 | b.d.l. | b.d.l. | 0.9840 | 100.0000 |
| 9 | P1-I-01-9 | b.d.l. | 0.0000 | 45.2830 | 53.8580 | b.d.l. | b.d.l. | 0.8590 | 100.0000 |
| 10 | P1-I-01-10 | 0.0170 | 0.0350 | 45.6730 | 53.7960 | 0.0110 | b.d.l. | 0.4680 | 100.0000 |
| 11 | P1-I-01-11 | 0.3110 | 0.0210 | 45.3530 | 53.2960 | 0.0450 | b.d.l. | 0.9740 | 100.0000 |
| 12 | P1-I-01-12 | b.d.l. | 0.0020 | 44.6510 | 53.7580 | 0.0160 | 0.0280 | 1.5460 | 100.0000 |
| 13 | P1-I-01-13 | b.d.l. | 0.0000 | 46.2290 | 53.7530 | 0.0180 | b.d.l. | b.d.l. | 100.0000 |
| 14 | P1-I-01-14 | b.d.l. | 0.0190 | 44.9780 | 53.7700 | 0.0570 | 0.0110 | 1.1650 | 100.0000 |
| 15 | P1-I-01-15 | 5.1530 | 0.0000 | 44.8280 | 49.9870 | b.d.l. | 0.0320 | b.d.l. | 100.0000 |
|  |  |  |  |  |  |  |  |  |  |
| **Point** | **Sample number** | **As (Atom)** | **Ni  (Atom)** | **Fe (Atom)** | **S  (Atom)** | **Au (Atom)** | **Cu (Atom)** | **Co (Atom)** | **Total (Atom)** |
| 1 | P1-I-01-1 | b.d.l. | 0.0735 | 32.2754 | 67.6500 | b.d.l. | 0.0012 | b.d.l. | 100.0001 |
| 2 | P1-I-01-2 | b.d.l. | 0.0048 | 32.5500 | 67.4344 | 0.0102 | 0.0006 | b.d.l. | 100.0000 |
| 3 | P1-I-01-3 | b.d.l. | 0.0077 | 32.2691 | 67.0131 | 0.0043 | 0.0075 | 0.6983 | 100.0000 |
| 4 | P1-I-01-4 | 0.0156 | 0.0198 | 32.6514 | 66.7927 | 0.0007 | 0.0030 | 0.5167 | 99.9999 |
| 5 | P1-I-01-5 | b.d.l. | 0.0181 | 33.0026 | 66.9744 | b.d.l. | b.d.l. | 0.0049 | 100.0000 |
| 6 | P1-I-01-6 | b.d.l. | 0.0613 | 32.8467 | 67.0705 | 0.0142 | 0.0073 | b.d.l. | 100.0000 |
| 7 | P1-I-01-7 | b.d.l. | 0.0638 | 33.0254 | 66.9108 | b.d.l. | b.d.l. | b.d.l. | 100.0000 |
| 8 | P1-I-01-8 | 0.2875 | b.d.l. | 31.6710 | 67.3756 | b.d.l. | b.d.l. | 0.6659 | 100.0000 |
| 9 | P1-I-01-9 | b.d.l. | b.d.l. | 32.3637 | 67.0546 | b.d.l. | b.d.l. | 0.5817 | 100.0000 |
| 10 | P1-I-01-10 | 0.0092 | 0.0237 | 32.6514 | 66.9961 | 0.0021 | b.d.l. | 0.3176 | 100.0001 |
| 11 | P1-I-01-11 | 0.1659 | 0.0139 | 32.5378 | 66.6104 | 0.0093 | b.d.l. | 0.6627 | 100.0000 |
| 12 | P1-I-01-12 | b.d.l. | 0.0014 | 31.9398 | 66.9898 | 0.0032 | 0.0174 | 1.0484 | 100.0000 |
| 13 | P1-I-01-13 | b.d.l. | b.d.l. | 33.0501 | 66.9464 | 0.0035 | b.d.l. | b.d.l. | 100.0000 |
| 14 | P1-I-01-14 | b.d.l. | 0.0125 | 32.1744 | 67.0045 | 0.0117 | 0.0071 | 0.7898 | 100.0000 |
| 15 | P1-I-01-15 | 2.8290 | b.d.l. | 33.0163 | 64.1339 | b.d.l. | 0.0208 | b.d.l. | 100.0000 |
