# Supplementary material for: Stromatolitic Mounds in Tidal‐Facies Sandstones of the Paleoarchean Moodies Group (Barberton Greenstone Belt, Eswatini)
Source: Geobiology. 2025 May 2;23(3):e70020. doi: 10.1111/gbi.70020 (PMC12047067; doi:10.1111/gbi.70020)
Supplement: Supplementary file 2 — Table S2: Paleotemperature calculations. [file GBI-23-e70020-s003.docx]

**S table 2 - Paleotemperature calculations using Raman Spectroscopy of Carbonaceous Matter, n = 45**

| Sample and spectra | |  | Peak parameters | |  |  | Temperatures [°C] | |
| --- | --- | --- | --- | --- | --- | --- | --- | --- |
|  |  | G | D1 | D2 | R2=(D1/(G+D1+D2) | | T= -445*R2+641 | |
| Sp 1: 19-117-P2-II | | 37341.50 | 31875.10 | 11496.80 | 0.394917077 | | 465.26 | |
| Sp 2: 19-117-P2-II | | 36491.00 | 26503.20 | 11181.70 | 0.357302035 | | 482.00 | |
| Sp 8: 19-117-P2-II | | 15620.40 | 6725.15 | 2360.09 | 0.272211123 | | 519.87 | |
| Sp 9: 19-117-P2-II | | 19373.10 | 12453.80 | 5905.77 | 0.330053505 | | 494.13 | |
| Sp 13: 19-117-P2-II | | 28305.60 | 13183.20 | 8289.00 | 0.264840953 | | 523.15 | |
| Sp 15: 19-117-P2-II | | 33936.90 | 19787.20 | 8713.61 | 0.316911046 | | 499.97 | |
| Sp 32: 19-117-P2-II | | 24808.60 | 17606.00 | 4882.07 | 0.37224608 | | 475.35 | |
|  | |  |  |  | Average | | 494.25 | |
|  |  |  |  |  |  | |  |  |
| Sp 1: 19-117-P2-VI | | 24115.80 | 8904.27 | 3314.06 | 0.245066278 | | 531.95 | |
| Sp 2: 19-117-P2-VI | | 70732.90 | 27907.60 | 14019.90 | 0.24771437 | | 530.77 | |
| Sp 3: 19-117-P2-VI | | 60607.30 | 44094.90 | 10387.80 | 0.383134069 | | 470.51 | |
| Sp 4: 19-117-P2-VI | | 36309.90 | 20820.10 | 6495.73 | 0.32722768 | | 495.38 | |
| Sp 5: 19-117-P2-VI | | 60730.40 | 27418.50 | 10120.50 | 0.27901361 | | 516.84 | |
| Sp 6: 19-117-P2-VI | | 37977.10 | 22497.70 | 5805.24 | 0.339434014 | | 489.95 | |
| Sp 9: 19-117-P2-VI | | 9605.13 | 4125.74 | 2139.00 | 0.259973144 | | 525.31 | |
| Sp 14: 19-117-P2-VI | | 43685.30 | 25571.60 | 6831.80 | 0.336076185 | | 491.45 | |
|  | |  |  |  | Average | | 506.52 | |
|  |  |  |  |  |  | |  |  |
| Sp 1: 21-214-I | | 49856.70 | 33362.20 | 10168.40 | 0.357245578 | | 482.03 | |
| Sp 2: 21-214-I | | 25247.30 | 12188.20 | 5633.94 | 0.282989516 | | 515.07 | |
| Sp 3: 21-214-I | | 50451.10 | 23122.70 | 7164.26 | 0.286391573 | | 513.56 | |
| Sp 4: 21-214-I | | 57312.70 | 25101.60 | 8062.98 | 0.277435396 | | 517.54 | |
| Sp 5: 21-214-I | | 18168.70 | 10553.60 | 3137.15 | 0.331254934 | | 493.59 | |
| Sp 6: 21-214-I | | 17513.40 | 6887.81 | 2617.53 | 0.254927136 | | 527.56 | |
|  | |  |  |  | Average | | 508.22 | |
|  |  |  |  |  |  | |  |  |
| Sp 1: 21-214-II | | 26985.00 | 16244.20 | 4552.54 | 0.33996669 | | 489.71 | |
| Sp 2: 21-214-II | | 23366.60 | 20522.60 | 2575.03 | 0.441686002 | | 444.45 | |
| Sp 24: 21-214-II | | 26171.50 | 17053.70 | 3360.75 | 0.366069598 | | 478.10 | |
| Sp 28: 21-214-II | | 28595.80 | 24895.30 | 2481.24 | 0.444778617 | | 443.07 | |
| Sp 29: 21-214-II | | 22525.40 | 14319.40 | 2449.49 | 0.36441427 | | 478.84 | |
| Sp 3: 21-214-II | | 27462.40 | 21030.40 | 7169.08 | 0.377824105 | | 472.87 | |
| Sp 30: 21-214-II | | 25584.50 | 9235.65 | 5537.02 | 0.228847811 | | 539.16 | |
| Sp 39: 21-214-II | | 32174.20 | 21390.20 | 7219.43 | 0.351906091 | | 484.40 | |
| Sp 51: 21-214-II | | 37742.60 | 27671.70 | 6900.61 | 0.382655527 | | 470.72 | |
| Sp 6: 21-214-II | | 24402.30 | 24019.10 | 5446.67 | 0.445887517 | | 442.58 | |
| Sp 7: 21-214-II | | 47089.10 | 31114.90 | 9520.59 | 0.354688463 | | 483.16 | |
|  | |  |  |  | Average | | 475.19 | |
|  |  |  |  |  |  |  |  |  |
|  |  |  |  |  |  |  |  |  |
|  |  |  |  |  |  |  |  |  |
| Sp 24: 21-214-II-1 | | 18742.40 | 12907.90 | 3741.93 | 0.364709994 | | 478.70 | |
| Sp 25: 21-214-II-1 | | 35561.20 | 22153.90 | 9998.85 | 0.327168922 | | 495.41 | |
| Sp 30: 21-214-II-1 | | 16272.50 | 7251.06 | 2088.72 | 0.283108728 | | 515.02 | |
| Sp 31: 21-214-II-1 | | 13696.00 | 9785.22 | 1515.09 | 0.39146658 | | 466.80 | |
| Sp 33: 21-214-II-1 | | 53989.20 | 21331.60 | 6999.81 | 0.259128303 | | 525.69 | |
| Sp 34: 21-214-II-1 | | 42220.90 | 23372.50 | 8862.65 | 0.313910018 | | 501.31 | |
|  | |  |  |  | Average | | 497.15 | |
|  |  |  |  |  |  |  |  |  |
| Sp 1: 21-214-II-2 | | 11325.10 | 5565.36 | 3926.24 | 0.267350733 | | 522.03 | |
| Sp 2: 21-214-II-2 | | 76953.50 | 36656.60 | 11003.90 | 0.29416117 | | 510.10 | |
| Sp 3: 21-214-II-2 | | 50417.50 | 28056.50 | 7079.45 | 0.327941188 | | 495.07 | |
| Sp 4: 21-214-II-2 | | 28042.30 | 13718.90 | 4868.83 | 0.294207402 | | 510.08 | |
| Sp 5: 21-214-II-2 | | 44671.00 | 13649.70 | 7845.42 | 0.2062944 | | 549.20 | |
| Sp 6: 21-214-II-2 | | 26067.10 | 13111.20 | 5287.65 | 0.294859325 | | 509.79 | |
|  | |  |  |  | Average | | 516.04 | |
|  |  |  |  |  |  |  |  |  |
|  |  |  |  |  |  |  |  |  |
| Average of all measurements | | | |  |  |  | 499.56 | |

| Sample and spectra |  | Peak parameters | |  | Temperatures [°C] |
| --- | --- | --- | --- | --- | --- |
|  | G | D1 | D2 | R2=(D1/(G+D1+D2) | T= -445*R2+641 |
|  |  |  |  |  |  |
| 21-207 1.1 | 9826.50 | 11029.20 | 1490.90 | 0.493551592 | 421.37 |
| 21-207 1.2 | 3782.20 | 4746.50 | 816.70 | 0.507896933 | 414.99 |
| 21-207 3.1 | 18553.60 | 18260.10 | 1319.23 | 0.478853841 | 427.91 |
| 21-207 3.2 | 17498.10 | 18140.50 | 1319.23 | 0.490843212 | 422.57 |
| 21-207 4.1 | 6664.30 | 8022.60 | 1427.70 | 0.497846673 | 419.46 |
| 21-207 6.1 | 6260.00 | 7506.20 | 999.00 | 0.508371035 | 414.77 |
| 21-207 6.2 | 8778.90 | 9502.40 | 1658.60 | 0.476552039 | 428.93 |
| 21-207 6.3 | 7695.60 | 8330.90 | 1136.40 | 0.485401651 | 425.00 |
| 21-207 8.1 | 8454.90 | 9599.30 | 1419.70 | 0.492931565 | 421.65 |
| 21-207 8.2 | 10127.10 | 12058.00 | 1452.30 | 0.510123787 | 413.99 |
| 21-207 8.3 | 6091.30 | 6958.60 | 1062.50 | 0.493084096 | 421.58 |
|  |  |  |  |  |  |
|  | | | | Average | 421.76 |
