# Supplementary material for: Stromatolitic Mounds in Tidal‐Facies Sandstones of the Paleoarchean Moodies Group (Barberton Greenstone Belt, Eswatini)
Source: Geobiology. 2025 May 2;23(3):e70020. doi: 10.1111/gbi.70020 (PMC12047067; doi:10.1111/gbi.70020)
Supplement: Supplementary file 3 — Figure S1: Outcrop documentation of calcareous structures at Site 2. Figure S2: Outcrop documentation of calcareous structures at Site 3. Figure S3: Description of sample 19‐117 — locations of plugs and thin sections. Figure S4: Description of sample 21‐214 — cm‐scale silicified and calcareous mounds. Figure S5: Morphological variation and composition of sample 19‐117‐P4. Figure S6: Description of wrinkly laminae at Site 1. Figure S7: Variation of cm‐scale silicified and calcareous stromatolites (sample 19–117). Figure S8: Possible small‐scale soft‐sediment deformation (sample 19–117‐α). Figure S9: Representative Raman spectra of Quartz, Dolomite, and Siderite. Figure S10: Three‐dimensionality of mini‐mounds visualized by serial slabbing of slab of sample 21–214. [file GBI-23-e70020-s001.pdf]

S figure 1 – Outcrop documentation of calcareous structures at Site 2

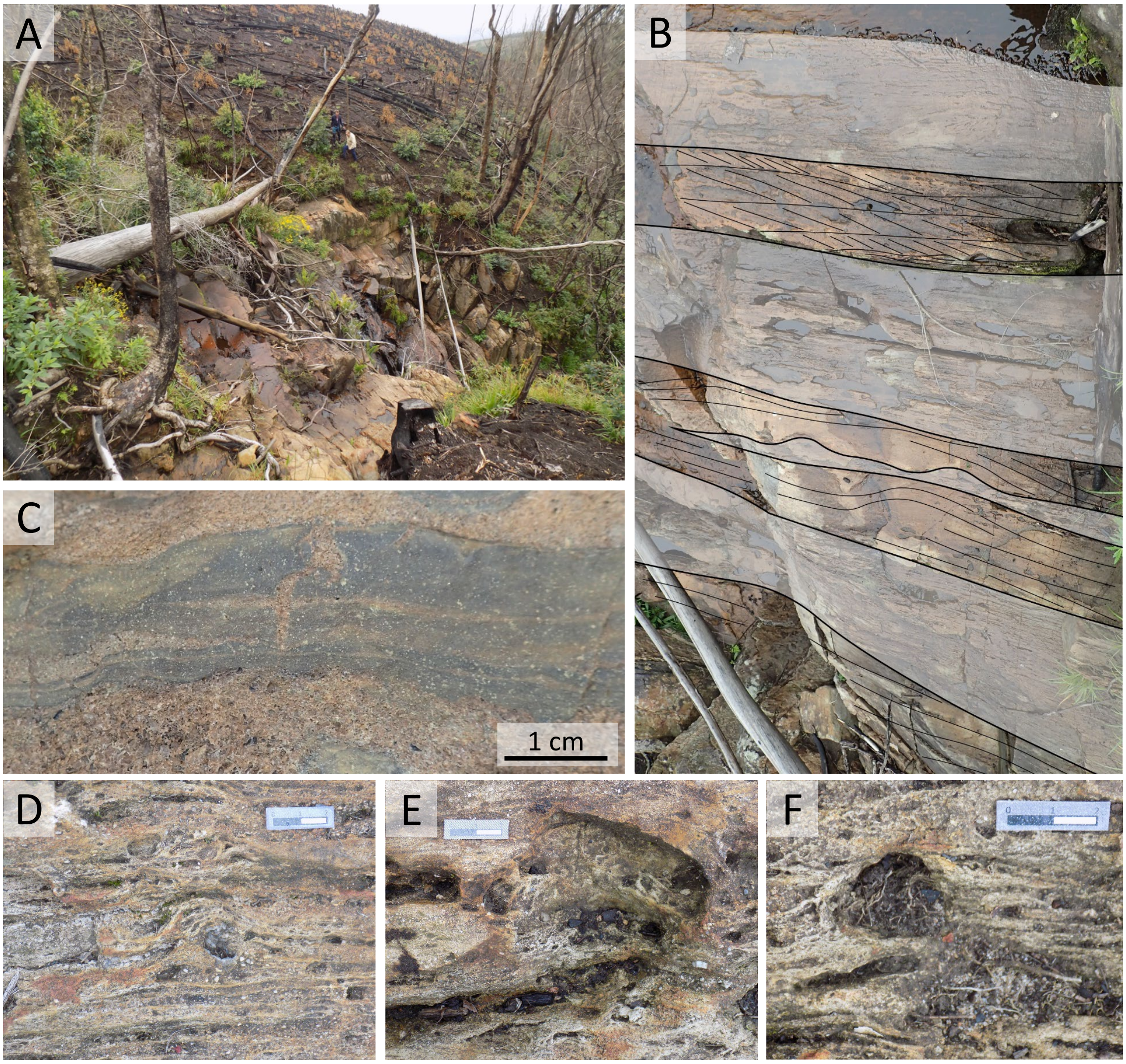

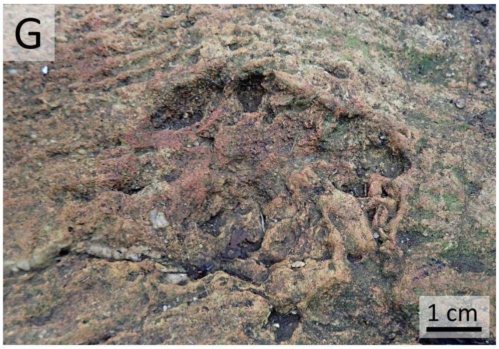

Site 2 is located in a gully (see Figs. 1, 2) within a pine plantation, exposing small calcareous mounds in Moodies Group sandstones, interbedded with up to 5 cm thick shale beds. A) Overview photograph across the drainage. Logging had recently taken place (year 2021). Persons (dashed circle) for scale. B) Outcrop photograph overlain by a translucent line drawing, illustrating the pattern of bundled calcareous laminae interbedded with medium- to coarse- grained sandstone. Section is ca. 50 cm thick. C) Compacted desiccation crack within shale, attesting to wetting-drying cycles. D - G) Weathered, cm-scale, internally structured calcareous mounds embedded in mostly horizontally stratified, partially granularly, poorly sorted medium-grained sandstone.

S figure 2 – Outcrop documentation calcareous structures at Site 3

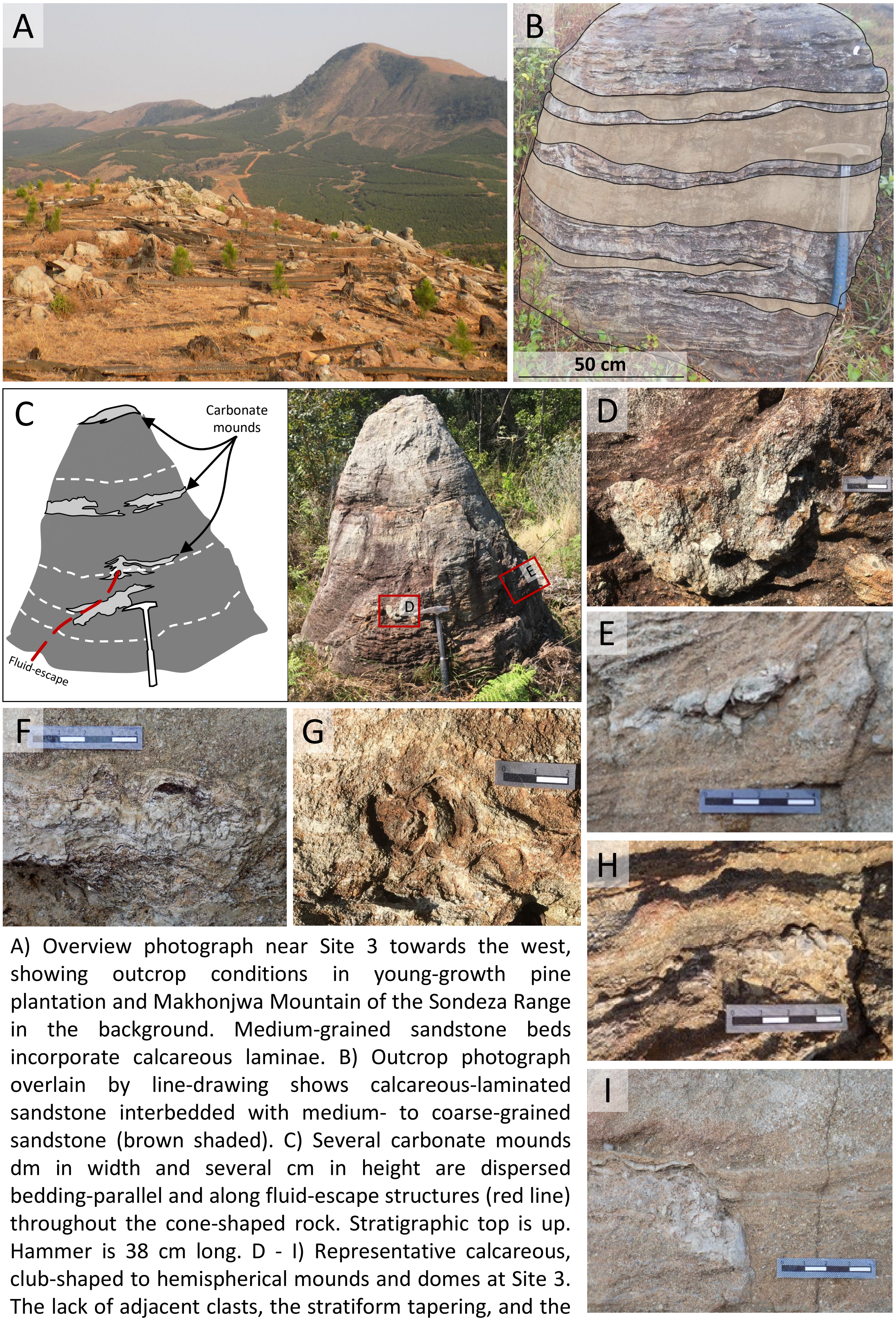

A) Overview photograph near Site 3 towards the west, showing outcrop conditions in young-growth pine plantation and Makhonjwa Mountain of the Sondeza Range in the background. Medium-grained sandstone beds incorporate calcareous laminae. B) Outcrop photograph overlain by line-drawing shows calcareous-laminated sandstone interbedded with medium- to coarse-grained sandstone (brown shaded). C) Several carbonate mounds dm in width and several cm in height are dispersed bedding-parallel and along fluid-escape structures (red line) throughout the cone-shaped rock. Stratigraphic top is up. Hammer is 38 cm long. D - I) Representative calcareous, club-shaped to hemispherical mounds and domes at Site 3. The lack of adjacent clasts, the stratiform tapering, and the gradation from calcareous to siliciclastic composition indicate that these structures are in place and were not transported.

S figure 3 – Description of sample 19-117 – locations of plugs and thin sections

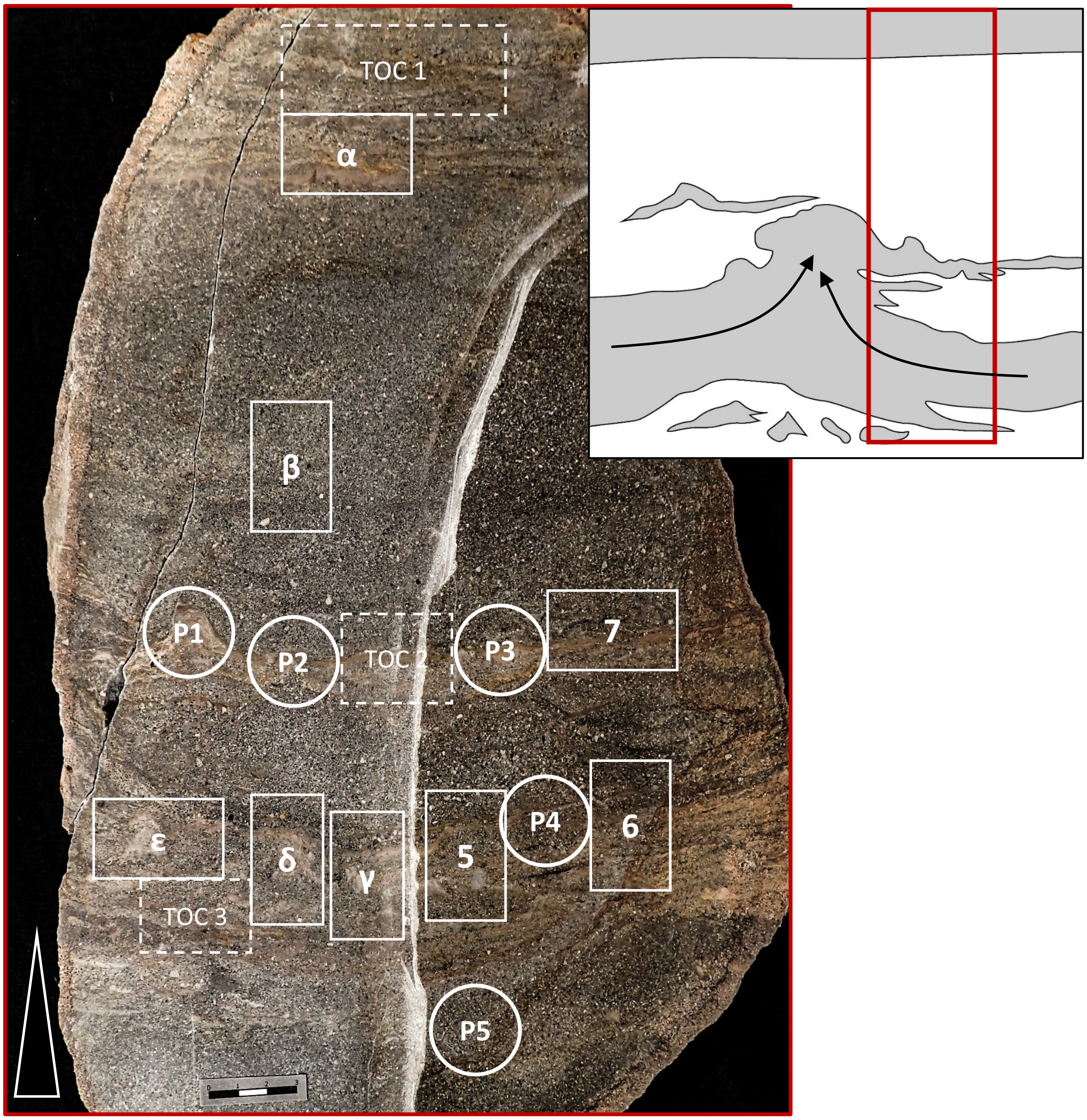

Sample 19-117, showing locations of thin-sections (white solid rectangles), plugs (white circles), and TOC measurements (dashed white squares). Red rectangle in the inset sketch (right) shows approximate location of the polished slab (left) compared to the substantially larger fluid-escape structure. The inset is further a schematical line drawing of the weathered outcrop surface, shown in Figure 3C. Black arrows indicate inferred direction of fluid flow. Thin-sections (Greek letters and numbers) and plugs (P) indicate a feldspathic litharenitc to subarkose composition.

Thin-section α (top) is described in detail in S figure 8. Other thin-sections and plugs were taken from two laminated and calcareous beds including small mounds. Their micro-scale structures are described in S figures 5 and 7. The upper bed is 1-3 cm thick, unevenly laminated, with isolated mounds up to 2.5 cm in height. The lower bed is ~7 cm thick and incorporates numerous domes and mounds, interbedded with fine- to medium-grained sandstone.

S figure 4 – Description of sample 21-214 – cm-scale silicified and calcareous mounds

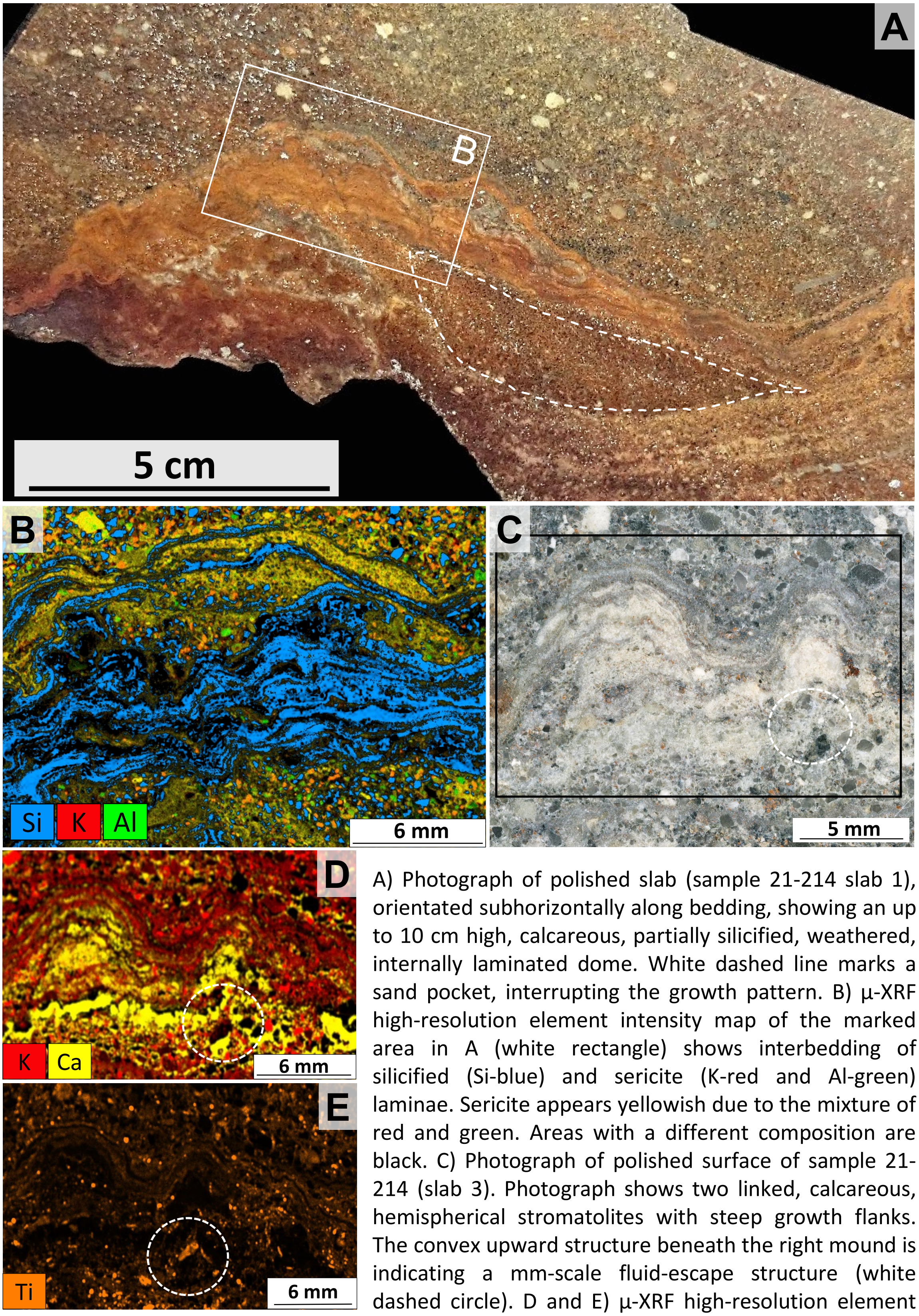

A) Photograph of polished slab (sample 21-214 slab 1), orientated subhorizontally along bedding, showing an up to 10 cm high, calcareous, partially silicified, weathered, internally laminated dome. White dashed line marks a sand pocket, interrupting the growth pattern. B) µ-XRF high-resolution element intensity map of the marked area in A (white rectangle) shows interbedding of silicified (Si-blue) and sericite (K-red and Al-green) laminae. Sericite appears yellowish due to the mixture of red and green. Areas with a different composition are black. C) Photograph of polished surface of sample 21-214 (slab 3). Photograph shows two linked, calcareous, hemispherical stromatolites with steep growth flanks. The convex upward structure beneath the right mound is indicating a mm-scale fluid-escape structure (white dashed circle). D and E) µ-XRF high-resolution element intensity maps of marked area in C (black rectangle), showing (D) K (red) and Ca (yellow), and (E) Ti. These elements are representing sericite (K), dolomite (Ca) and rutile/anatase (Ti).

S figure 5 – Morphological variation and composition of sample 19-117-P4

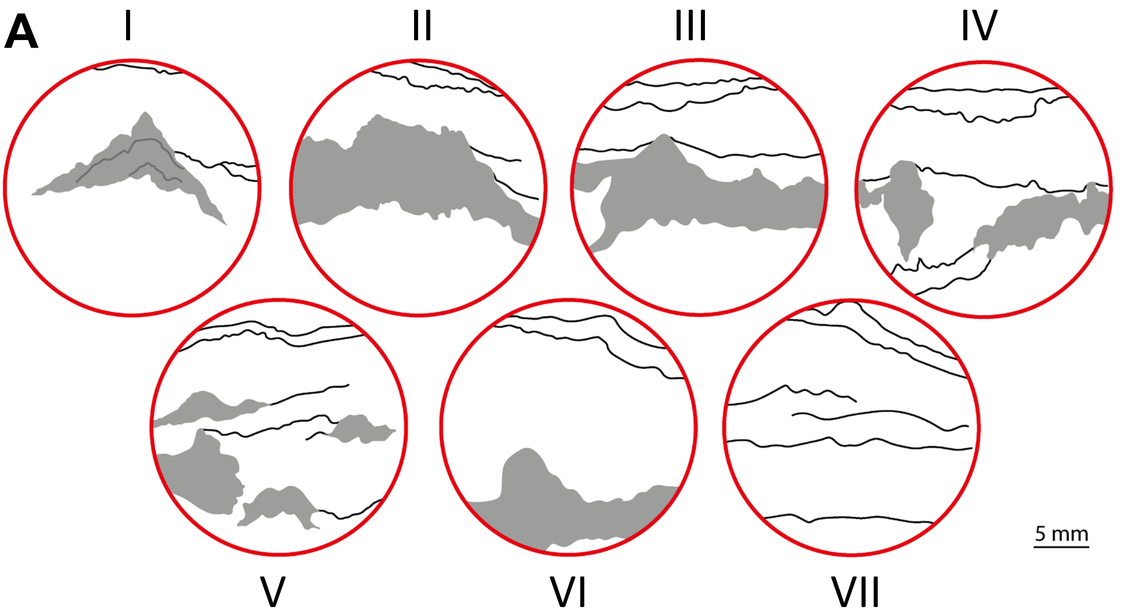

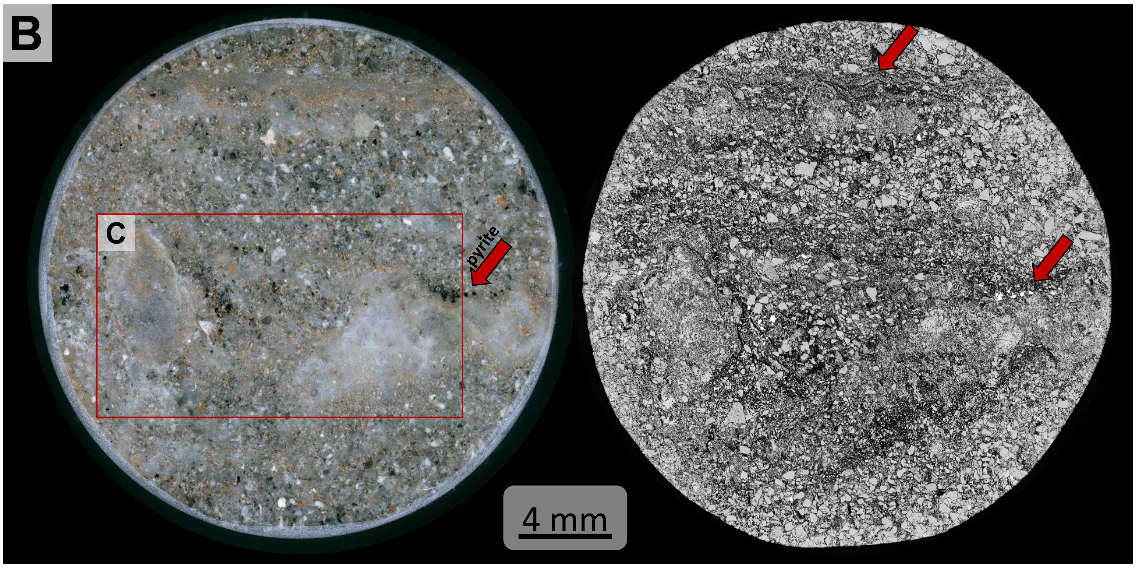

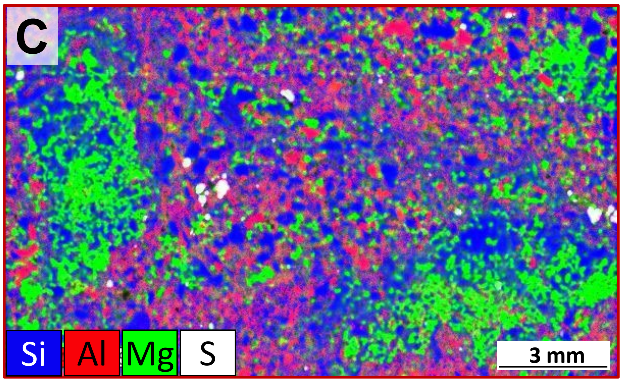

A) Serial slabbing of Plug 19-117-P4. showing the in-depth transition of carbonate bodies (grey) and wrinkle lamination (black lines), within a length of ~10 cm (from slice I to VII). Slice I shows a cone shaped mound with internal lamination (grey lines). The carbonate structures shown in slice II to VI lack distinct internal lamination. The mounds show a strong morphological variation. B) Photographs of a polished slice IV under plain reflected light (left) and coaxial reflected light (right). Both show two adjacent calcareous mounds lacking internal lamination. Bedding is subhorizontal, indicated by laminae at the top of the section and by a pyrite-rich bed along the right flank of the right mound (see red arrows). C) µ-XRF high-resolution element intensity map of the section marked in A (red rectangle), representing silica (Si), sericite (Al), dolomite (Mg), and pyrite (S). A preferred orientation of the Mg-rich (green; likely dolomite) crystals is not apparent.

S figure 6 – Description of wrinkly laminae at Site 1

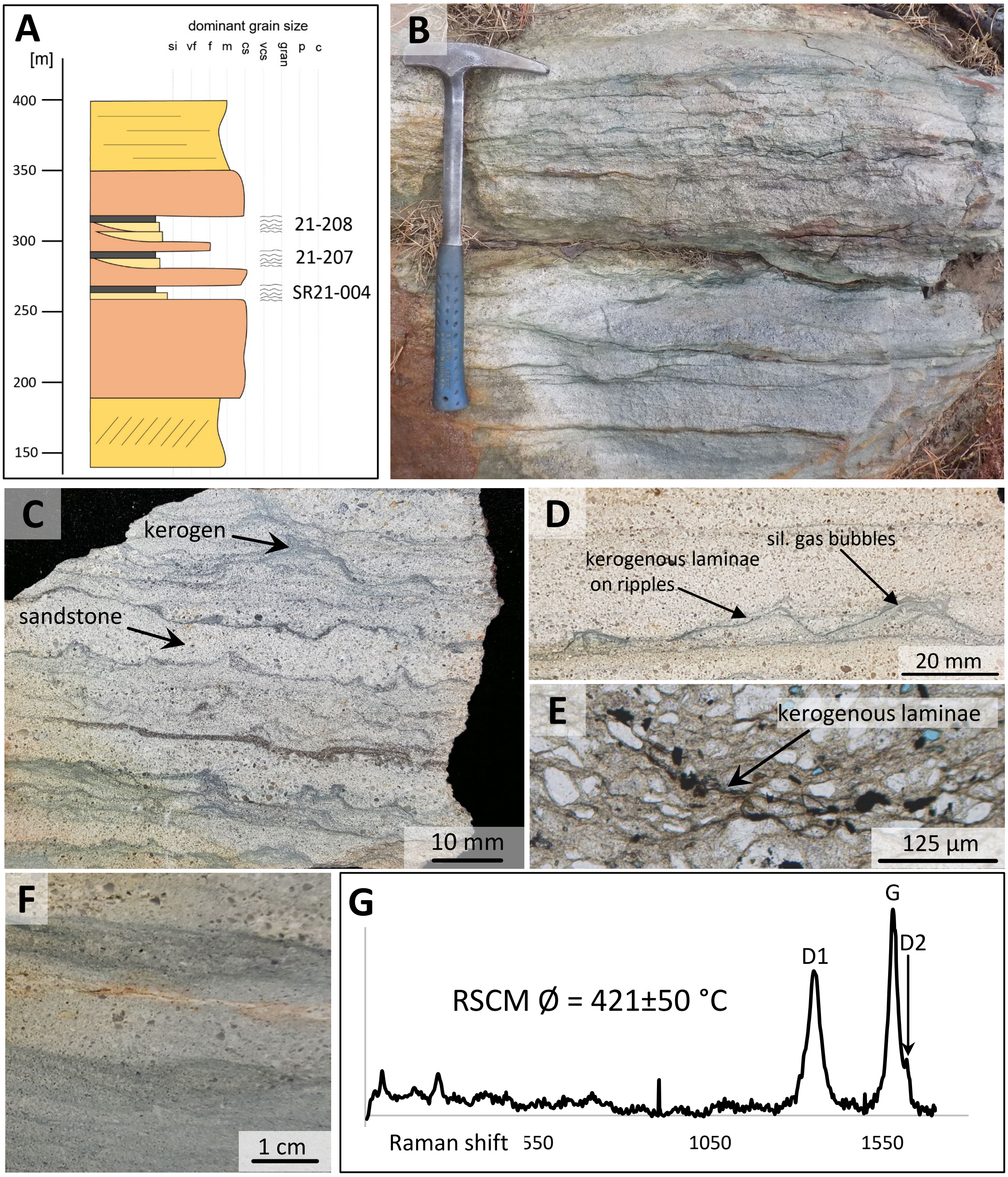

A) Detailed stratigraphic column of Site 1 showing three fine-grained beds and the location of three samples; for legend see Fig. 2. B) Outcrop photograph of wrinkled and tufted kerogenous laminae within in fine-grained tuffaceous planar and rippled sandstone, interbedded with gravel free coarse-grained sandstone. Hammer for scale is 44 cm long. C) Hand-sample photograph of a polished section (sample 21-207) showing kerogen-rich laminae interbedded with fine- to medium-grained sandstone. D) Close-up photograph of polished section (sample 21-208). Laminae show wrinkles, tufts, and silicified gas bubble fill overgrowing ripples in cross section. E) Thin-section photomicrograph of dark opaque laminae wrapping around sand grains. Laminae consist of kerogen and metal oxides, e.g., rutile (sample 21-207) within a sericite matrix. F) Close-up photograph of polished section (sample SR21-004). This sample contains up to 5 cm thick beds of condensed dark laminae interbedded with shaly silt. G) Representative Raman spectrum of kerogen of sample 21-207 showing well-developed D- and G-peaks of organic carbon. The sample shows an average metamorphic peak temperature of 421±50 °C.

S figure 7 – variation of cm-scale silicified and calcareous stromatolites (sample 19-117)

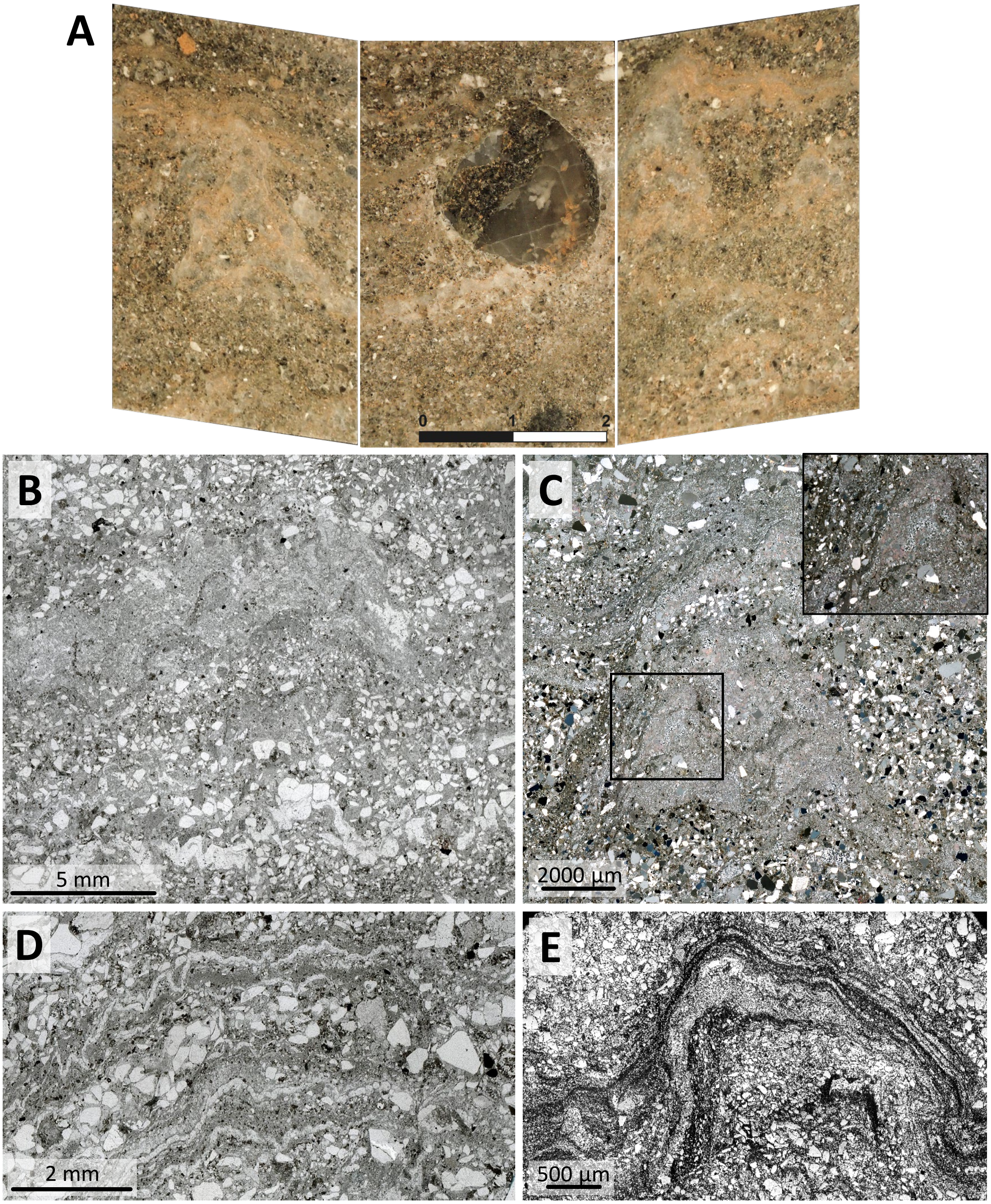

Detailed analysis of sedimentary structures at micro-scale from sample 19-117 (S figure 3). A) 3-D panorama of three polished thick-section faces (19-117-δ). Faces were originally oriented perpendicular to each other. They show a carbonate mound adjacent to a well-rounded black chert clast approx. 2 cm in diameter. B) Thin-section photomicrograph (19-117–5, plain light) of an undulous lamina of microquartz (below) overlain by convex-up, internally unstructured calcareous mounds outlined by heavy minerals. C) Thin-section photomicrograph of an internally structured, calcareous (reddish) and siliceous (grey) conical mound (19-117–ε, crossed nicols). Bands of microquartz resemble silicified microbial laminae (e.g., see inset). D) Thin-section photomicrograph of several undulous laminae of microquartz (light grey) interbedded with sericite (dark grey) and quartzose sand grains (white); (19-117–5, plain light). E) Thin-section photomicrograph (19-117-P2-III, coaxial light) of a laminated, domical, calcareous stromatolite with steep margins.

S figure 8 – possible small scale soft sediment deformation
(sample 19-117-α of site 3)

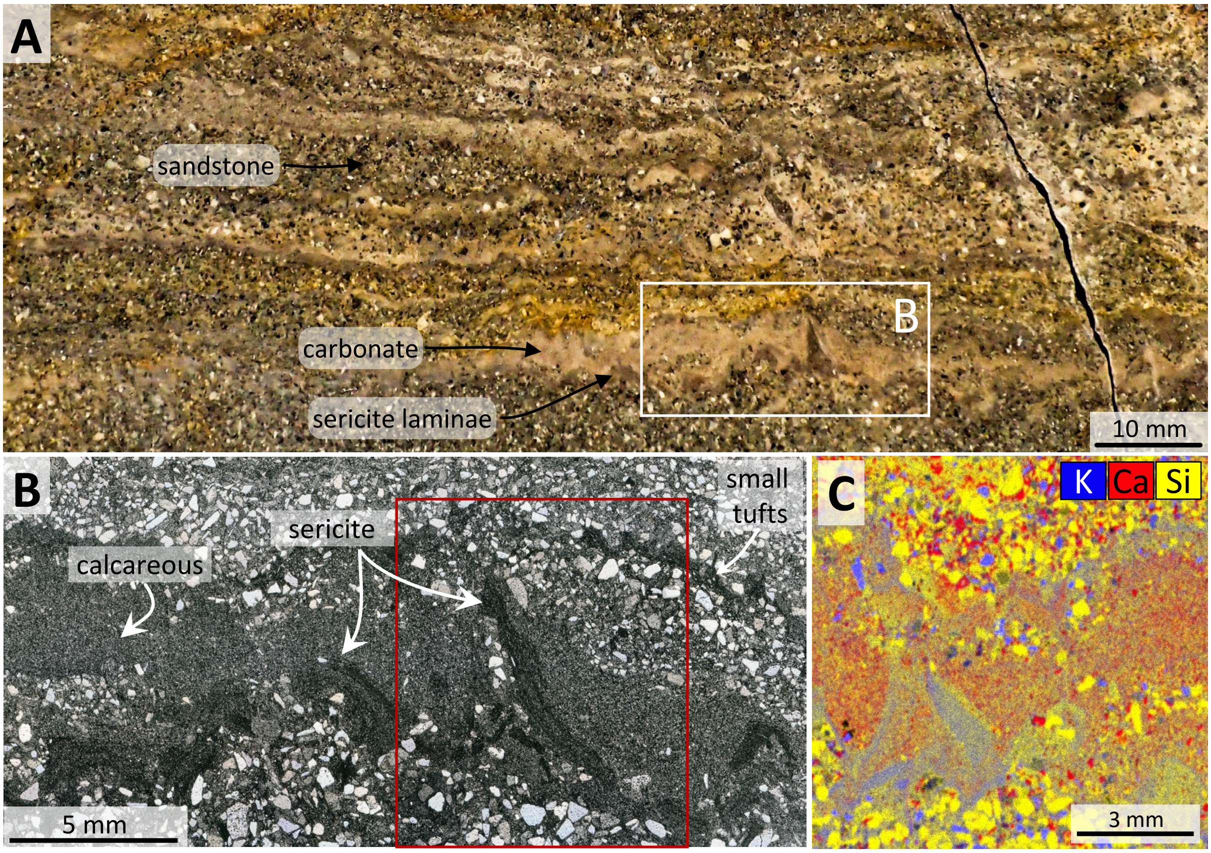

Laminated calcareous sandstones composed of sericite, carbonate, and fine- to medium-grained quartzose sandstone overlie the principal fluid-escape structure of sample 19-117; location shown in S figure 3. They show small-scale tufts and possible flame structures (see B). A) Close-up photograph of polished surface showing a set of undulous, carbonaceous, sericitic laminae within a fine- to medium-grained feldspathic litharenite. B) Photomicrograph of polished thin section (coaxial reflected light) of area shown in (A). Possibly carbonaceous sericitic laminae (dark grey) form tufts and domes overlying sandy sediment within a very fine-grained carbonate matrix, interspersed with microquartz. C) µ-XRF high-resolution element intensity map of the area marked in B by red rectangle, showing silica (Si), sericite (Al), and dolomite (Ca). The higher concentration of K within the tuft contrasts with the higher Ca concentration in the surrounding matrix.

S figure 9 – Representative Raman spectra of Quartz, Dolomite, and Siderite

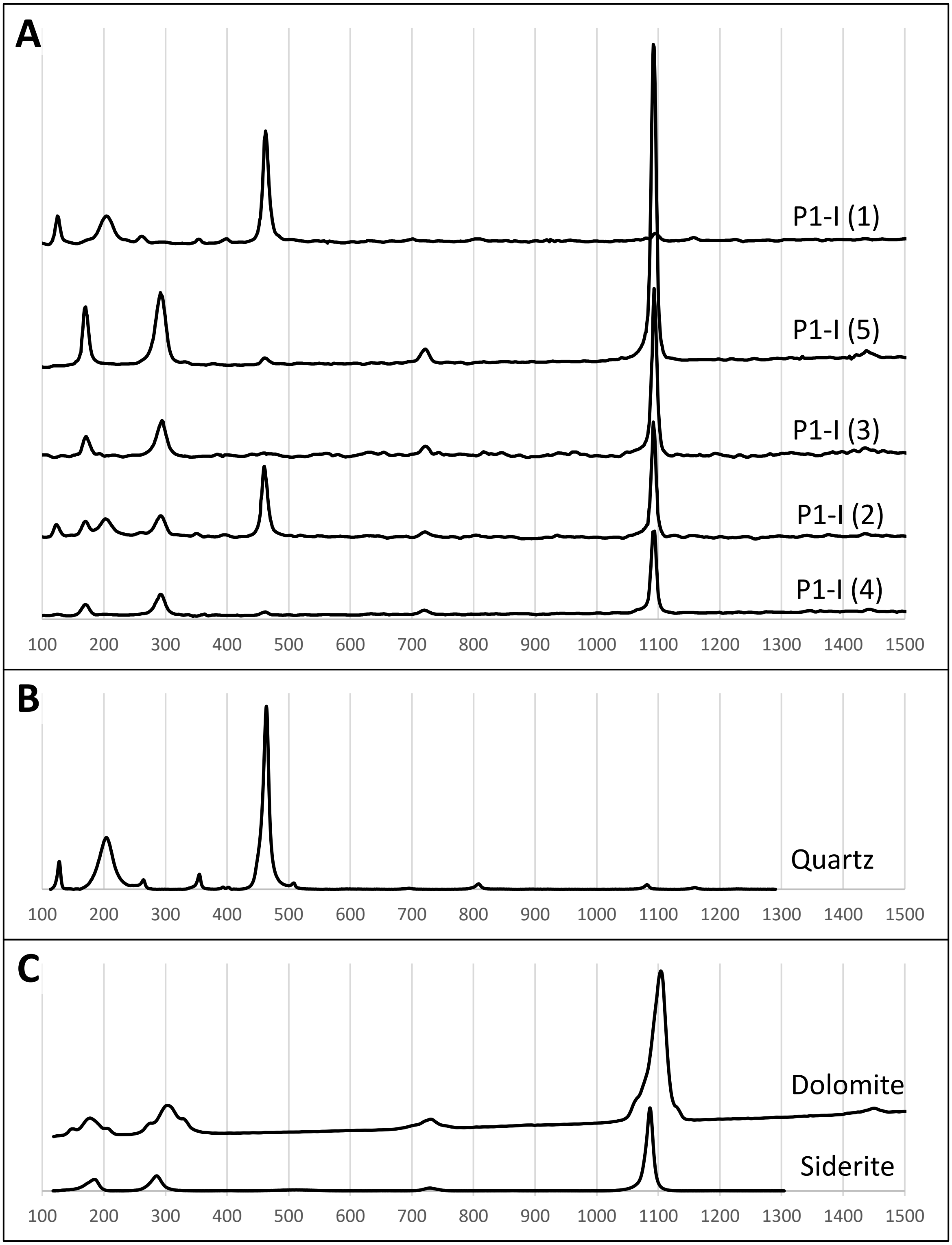

Representative Raman spectra (sample 19-117-P1-I, laser wavelength of 532 nm) showing spectra characteristic of dolomite mixed with quartz (A). (B) and (C) show type spectra of quartz, dolomite and siderite from the RUFF database (Armbruster et al., 2016).

S figure 10 – Three-dimensionality of mini-mounds visualized by serial slabbing of slab of sample 21-214

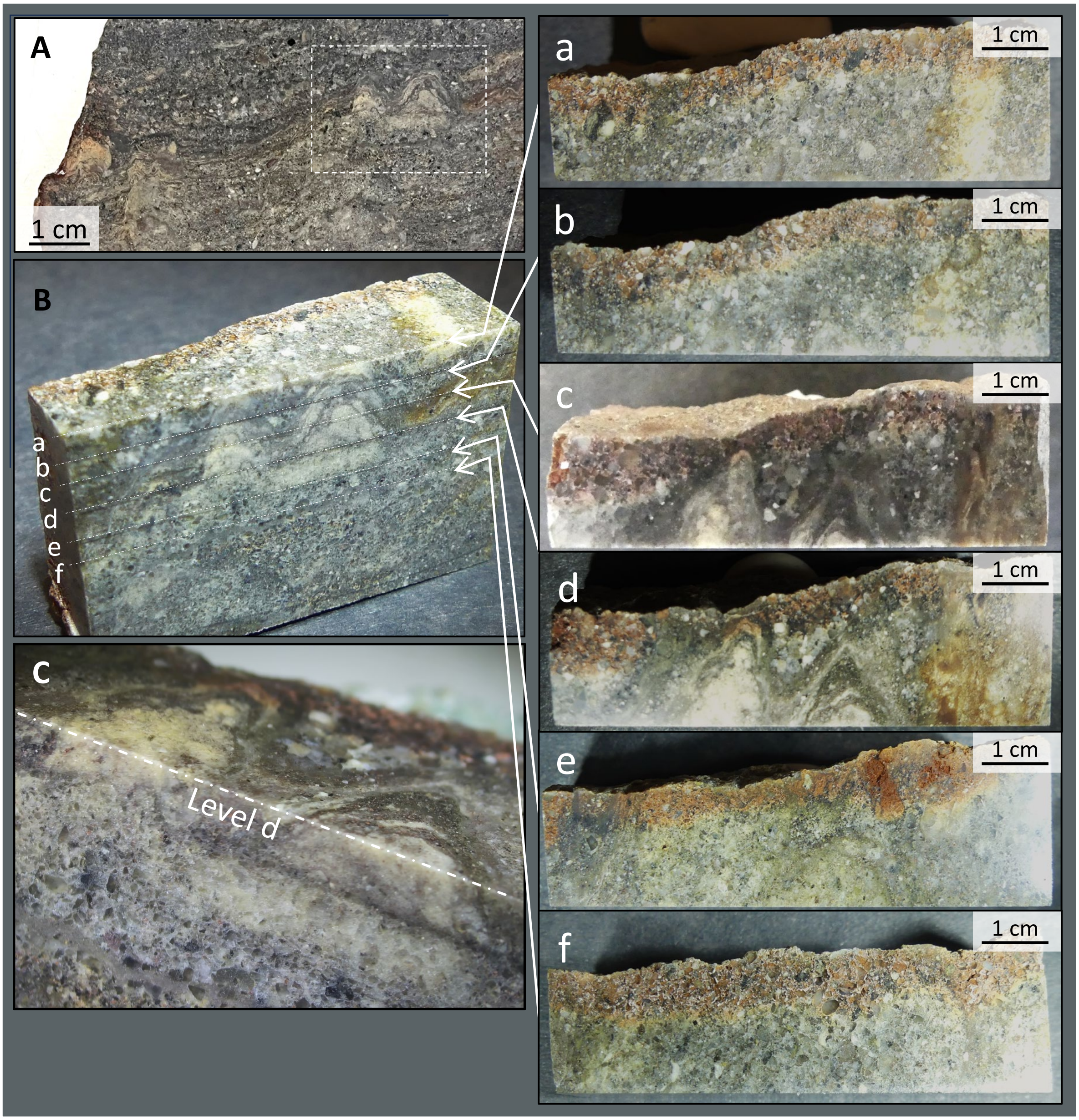

Three-dimensionality of mini-mounds visualized by serial slabbing of part of a ca. 10 mm thick slab of sample 21-214. (A) Photograph of polished hand sample showing the mounds (grey) and laminae (black). Area outlined by white dashed rectangle was cut out (see B). It includes two small linked mounds with flat base and domal tops within thinly bedded sandstone. (B) Oblique view of sample prior to serial slabbing (left), and top-down map views (a to f, right) of six serial slabs. Levels c and d show conical to wedge shapes and internal lamination. Level e shows indistinctly the oval base of a mound. Levels a, b, and f show the texture of under- and overlying sandstone, respectively, lacking traces of sedimentary or deformational structures. (C) Oblique frontal view of level d, showing the consistent geometrical relationships between vertical and horizontal transects. Horizontal black line are pencil marks, subparallel to bedding. White dashed line indicates the rectangular edge.

The images demonstrate that the mounds are distinct from primary sedimentary structures, such as flame structures or sand volcanoes. They also differ from tectonic structures, such as boudinage or folds, due to their 3-D shape. Instead, the mounds display characteristics consistent with their biogenic origin: They occur in specific beds within a well-preserved shallow-marine sequence, possess internal laminations, display flat bases and coniform to domal tops, and are made primarily of dolomite with stable-isotope values consistent with precipitation from biologically-influenced shallow water.
